# From Innovation to Application: Can Emerging Imaging Techniques Transform Breast Cancer Diagnosis?

**DOI:** 10.3390/diagnostics15212718

**Published:** 2025-10-27

**Authors:** Honda Hsu, Kun-Hua Lee, Riya Karmakar, Arvind Mukundan, Rehan Samirkhan Attar, Ping-Hung Liu, Hsiang-Chen Wang

**Affiliations:** 1 Division of Plastic Surgery, Dalin Tzu Chi Hospital, Buddhist Tzu Chi Medical Foundation, No. 2, Minsheng Road, Dalin, Chiayi 62247, Taiwan; hondahsu@yahoo.com.tw; 2 Department of Trauma, Changhua Christian Hospital, Changhua, No. 135, Nanxiao St., Changhua City 50006, Taiwan; 88847@cch.org.tw; 3 Department of Mechanical Engineering, National Chung Cheng University, 168, University Rd., Min Hsiung, Chia Yi 62102, Taiwan; karmakarriya345@gmail.com (R.K.); arvindmukund96@gmail.com (A.M.); 4 School of Engineering and Technology, Sanjivani University, Singnapur, Kopargaon 423603, Maharashtra, India; 5 Department of Computer Engineering, Sanjivani College of Engineering, Kopargaon 423603, Maharashtra, India; rehanattar6541@gmail.com; 6 Division of General Surgery, Kaohsiung Armed Forces General Hospital, 2, Zhongzheng 1st. Rd., Lingya District, Kaohsiung City 80284, Taiwan

**Keywords:** breast cancer, optical coherence tomography, Raman spectroscopy, photoacoustic imaging, hyperspectral imaging, contrast-enhanced spectral mammography, multispectral imaging, convolutional neural network

## Abstract

**Background/Objectives**: Breast cancer (BC) has emerged as a significant threat among female malignancies, resulting in approximately 670,000 fatalities. The capacity to identify BC has advanced over the past two decades because of deep learning (DL), machine learning (ML), and artificial intelligence. The early detection of BC is crucial; yet, conventional diagnostic techniques, including MRI, mammography, and biopsy, are costly, time-intensive, less sensitive, incorrect, and necessitate skilled physicians. This narrative review will examine six novel imaging approaches for BC diagnosis. **Methods**: Optical coherence tomography (OCT) surpasses existing approaches by providing non-invasive, high-resolution imaging. Raman Spectroscopy (RS) offers detailed chemical and structural insights into cancer tissue that traditional approaches cannot provide. Photoacoustic Imaging (PAI) provides superior optical contrast, exceptional ultrasonic resolution, and profound penetration and visualization capabilities. Hyperspectral Imaging (HSI) acquires spatial and spectral data, facilitating non-invasive tissue classification with superior accuracy compared to grayscale imaging. Contrast-Enhanced Spectral Mammography (CESM) utilizes contrast agents and dual energy to improve the visualization of blood vessels, enhance patient comfort, and surpass standard mammography in sensitivity. Multispectral Imaging (MSI) enhances tissue classification by employing many wavelength bands, resulting in high-dimensional images that surpass the ultrasound approach. The imaging techniques studied in this study are very useful for diagnosing tumors, staging them, and guiding surgery. They are not detrimental to morphological or immunohistochemical analysis, which is the gold standard for diagnosing breast cancer and determining molecular characteristics. **Results**: These imaging modalities provide enhanced sensitivity, specificity, and diagnostic accuracy. Notwithstanding their considerable potential, the majority of these procedures are not employed in standard clinical practices. **Conclusions**: Validations, standardization, and large-scale clinical trials are essential for the real-time application of these approaches. The analyzed studies demonstrated that the novel modalities displayed enhanced diagnostic efficacy, with reported sensitivities and specificities often exceeding those of traditional imaging methods. The results indicate that they may assist in early detection and surgical decision-making; however, for widespread adoption, they must be standardized, cost-reduced, and subjected to extensive clinical trials. This study offers a concise summary of each methodology, encompassing the methods and findings, while also addressing the many limits encountered in the imaging techniques and proposing solutions to mitigate these issues for future applications.

## 1. Introduction

Breast Cancer (BC) is among the most prevalent forms of cancer affecting women globally [1]. It is expanding more swiftly in developing nations than in developed ones [2]. According to the World Health Organization’s mortality statistics, there were 2.3 million new cases and 670,000 deaths in 2022, with an annual increase rate of 1% to 5% [3]. In 2020, the International Agency for Research on Cancer, part of the World Health Organization, declared that BC has officially become the most prevalent cancer worldwide [4].

The staging system for breast cancer is the globally acknowledged Tumor, Node, Metastasis (TNM) classification system, endorsed by guidelines such as NCCN and ESMO. The tumor stage is determined by three primary factors: T (size and location of the primary tumor), N (regional lymph node involvement), and M (absence or presence of metastasis in other areas). Non-invasive breast cancer, referred to as carcinoma in situ (DCIS or LCIS), should not be conflated with invasive carcinoma, which involves the proliferation of tumor cells beyond the basement membrane. The nodal status (N0-N3) and the presence of metastases (M0 or M1) can be utilized to further stratify invasive breast cancers. The accuracy of staging is crucial as it directly influences prognosis and guides the selection of appropriate treatments, including surgery, systemic therapy, and radiotherapy [5,6]. Morphological examination is still the main method of malignant tumor diagnosis. Radiologists, pathologists, and surgeons find it harder to make a diagnosis because there are so many BC patients. They need to know the type and stage of the tumor in order to plan the best treatment. In the past, imaging techniques like mammography, ultrasound, and MRI as well as histopathological analysis of biopsy specimens have been used to diagnose diseases. These methods are still the best way to confirm that a disease is cancerous. However, these traditional methods often face challenges stemming from observer variability, significant workloads, and the complexities involved in detecting early or subtle lesions. Digital pathology, machine learning, and deep learning algorithms are some of the new technologies that have come together in the last few years. These technologies are now a powerful tool that can help doctors find and classify breast cancer more accurately, quickly, and fairly [7].

Optical coherence tomography (OCT) is an imaging technique that produces high-resolution images with near-infrared light, resulting in detailed images comparable to ultrasound images [8]. This procedure is less unpleasant as it does not involve the insertion of devices into the body or any incisions to the skin [9]. OCT generates images that are crisper and sharper than those produced by conventional BC imaging modalities such as mammography and ultrasound. It was first developed to facilitate the management of BC via a non-destructive high-resolution imaging technique for the examination of tumor morphology [10].

The manual diagnosis of BC from mammography pictures is time-consuming and requires significant effort to identify and classify the type of cancer present [11]. In the initial stage, the likelihood of survival is enhanced, but in the advanced stage, the survival prospects for a BC patient diminish. Women over 40 should consistently attend the hospital for breast screenings, as diagnoses often occur at advanced stages due to neglect in self-examinations and clinical assessments [12]. Numerous robust models such as ResNet, GoogLeNet, and VGG have been developed over the past decade. Deep learning (DL) is a prevalent technique in picture segmentation and object recognition, significantly aiding in the detection of cancer images [13]. BC can be detected using many technologies, including X-ray scans, ultrasound imaging, Computed Tomography scans, and thermography [14]. Currently, many approaches, including OCT, Raman Spectroscopy (RS), Photoacoustic Imaging (PAI), Hyperspectral Imaging (HSI), Contrast-Enhanced Spectral Mammography (CESM), Multispectral Imaging (MSI), are emerging that demonstrate enhanced efficiency in the identification of BC.

In recent years, RS has cultivated a significant interest in cancer research and surgical procedures. It has been utilized to investigate and identify tumors in the brain, breast, lung, skin, uterus, and digestive tract, both in laboratory samples and during surgical procedures [15]. RS is extremely sensitive to minor molecular and structural alterations in tissues, making it effective for diagnosing biochemical changes in those tissues. RS does not obliterate the sample and supplies information regarding biomolecular alterations, and it is unnecessary to label or stain the tissue before detection [16].

Photoacoustic spectrum imaging is a developing, novel, and non-invasive technique utilized for cancer detection. It identifies specific molecules in tissues based on their light absorption properties and can indicate their composition and physical attributes [17]. When a tissue sample is pulsed or modulated with light at specified wavelengths, the absorbed light energy induces fast heating, leading to small tissue expansion that generates measurable pressure waves, thus yielding information about the sample [18]. This approach enables real-time mapping of tumor oxygenation. It detects internal light-absorbing chromophores such as hemoglobin and generates a three-dimensional parametric map of tissue oxygen saturation [19].

HSI, digital imaging is integrated with spectroscopy. Each pixel of the image is recorded and encompasses the reflected intensity for a distinct range of colors referred to as a spectrum [20]. HSI simultaneously captures both spatial and spectral information. Narrowly spaced wavelength bands are captured, subsequently leading to the construction of a detailed 3D dataset that illustrates the unique spectral fingerprints of distinct tissues [21]. HSI offers numerous advantages since it is non-invasive, non-contact, rapid, and does not require contrast chemicals or expose individuals to radiation [22].

In 2001, Lewin et al. proposed CESM, which enhances mammography by the successful use of intravenous contrast enhancement [23]. CESM distinguishes itself as a comprehensive method relative to other techniques, since it concurrently provides mammogram-like images alongside contrasting images that yield data on the neo-angiogenesis of lesions [24]. It has established itself as a unique and therapeutically valuable technique for early breast evaluation [25].

MSI is one of the spectrum imaging techniques that captures reflection or radiation data from an object using a defined and limited number of spectral bands, encompassing visible and near-infrared light. The reflectance or radiance of the target in that band, which contributes to the spectral information database, is documented [26]. He methodology typically relies on spectral bandwidths in the ultraviolet, visible, and infrared ranges to obtain spatial and spectral data from the objects being assessed [27].

There are many well-known problems with traditional diagnostic and morphological methods for breast cancer, even though they are widely used. Mammography and ultrasound are less accurate in women with dense breasts, and they can give false-positive results, which can lead to unnecessary biopsies and anxiety for the patient. Also, mammography exposes the patient to ionizing radiation. MRI is very sensitive and costly, takes a long time, and is not available in all clinical settings. Histopathological diagnosis remains the gold standard; however, this method is invasive due to biopsy sampling, carries a risk of sampling error, and is subject to inter-observer variability among pathologists. All these problems show how important it is to create and test new imaging methods that can either add to or improve existing ones and improve early diagnosis, diagnostic accuracy, and patient outcomes. This study discusses six distinct imaging modalities utilized for the detection of BC. The document comprises various sections, including methodology and a concise overview of each approach, detailing its history, principles, advantages, limitations, and applications. Each strategy is accompanied by case studies that detail the methodology, dataset utilized, and results obtained. The limitations section outlines the constraints of each technique, while the solutions part provides remedies for these limitations. Ultrasound, mammography, CT, and MRI are essential diagnostic modalities in oncology for identifying the primary tumor and assessing disease dissemination. However, the diagnosis of breast cancer and the determination of its molecular subtype are feasible solely following the morphological and immunohistochemical analysis of tumor tissue. The new imaging techniques discussed in this review, such as OCT, RS, PAI, HSI, CESM, and MSI, can be seen as useful diagnostic tools that can be used with morphological tools but not instead of them. They might help with early diagnosis, characterizing lesions, and evaluating surgical margins, but they cannot replace the diagnostic pathway that is already in place. This review adds to the literature by providing in-depth analyses of six novel breast imaging modalities that are not yet widely utilized in clinical practice but possess significant potential for early detection, lesion characterization, and intraoperative guidance. It discusses the pros and cons of each method in terms of how easy it is to use in a clinical setting, how comfortable it is for patients, how easy it is to get to, and how well it fits into the workflow. It identifies the evidence gaps, meaning that there are different types of studies, no standardization of endpoints, and not enough large-scale clinical validation, which shows that the current evidence base is weak. It also discusses where each of the modalities would fit best into current clinical regimens and what needs to be done to make them more widely used, while emphasizing the necessity to standardize and implement multicenter trials, as well as to integrate AI and radiomics, in order to maximize the clinical utility of these technologies. The paper aims to bridge the gap between promising research-level performance and actual clinical application, thereby offering a more comprehensive framework for future studies and clinical translation.

## 2. Literature Search Strategy

Relevant studies on BC were obtained by a comprehensive search of several scientific databases. Examples include ScienceDirect, ResearchGate, and Google Scholar, among others. It employs diverse combinations of keywords and various medical terminologies pertinent to BC, its diagnosis, treatments, and the array of imaging techniques utilized for detection, to identify the most relevant and suitable research publications.

### Inclusion and Exclusion Criteria for Review Article

**Figure 1:**
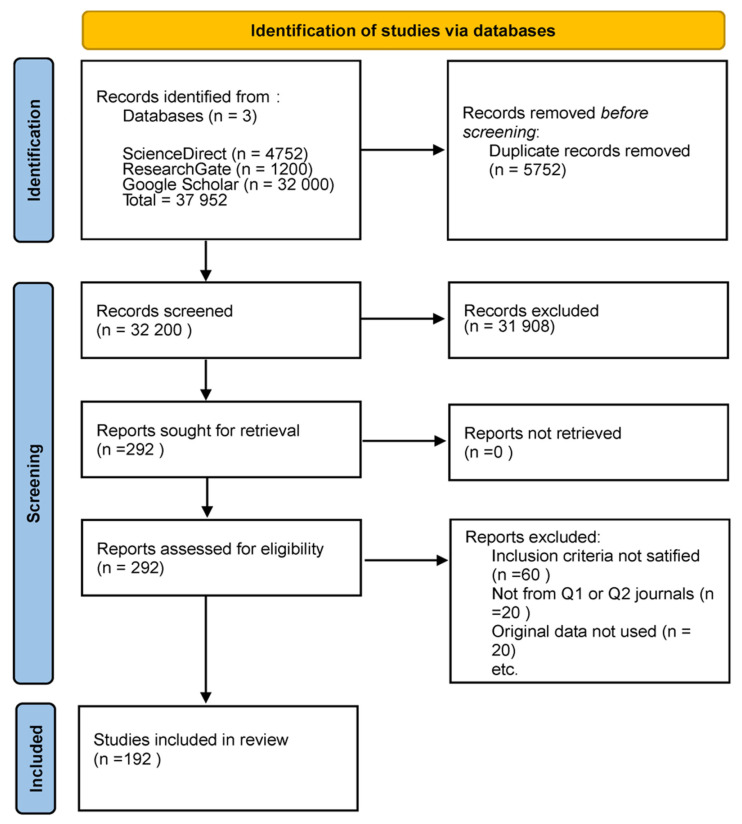
PRISMA flow diagram of the literature search and selection process. The figure shows the total number of studies retrieved from databases, the number excluded after screening and eligibility assessment, and the final number of studies included in this narrative review.

This review exclusively comprised original research studies utilizing human subjects and incorporating one or more of the emerging imaging methodologies: OCT, RS, PAI, HSI, CESM, or MSI. This review was conducted in accordance with the Preferred Reporting Items for Systematic Reviews and Meta-Analyses (PRISMA) guidelines. A comprehensive search was performed in PubMed, Scopus, Web of Science, and IEEE Xplore for articles published between January 2019 and December 2024 that appeared in peer-reviewed journals of scientific merit, primarily those classified as Q1 or Q2, with an H-index of 75 or greater. Research was necessary to demonstrate adherence to ethical standards, including obtaining institutional approval for human subject research. The excluded materials comprised case reports, editorials, review articles, meta-analyses, and conference abstracts. We excluded studies that lacked methodological detail, did not provide original imaging data, or utilized exceedingly small sample sizes. Nonetheless, in certain circumstances, less stringent thresholds were incorporated when pertinent due to their methodological contribution or the limited evidence available in a specific modality. This adaptability offered a comprehensive overview while maintaining methodological rigor. Figure 1 shows the PRISMA flow diagram, the literature identification and screening selection process, detailing the number of retrieved records, the reasons for exclusion during screening and eligibility assessments, and the final sample of studies incorporated in this review.

While the inclusion criteria primarily focused on human-based clinical trials, it was also consulted specific preclinical and technical trials as necessary. The rationale for incorporating these studies is that certain imaging modalities examined in this research have not been fully translated into clinical practice, and preclinical studies may represent the sole evidence regarding the device’s performance, reconstruction limitations, or functionality. Direct harmonization of endpoints across the included studies was not feasible due to significant design heterogeneity. Some studies reported results on a per-patient basis, while others analyzed outcomes on a per-patch or per-lesion basis. In vivo and ex vivo studies differed in their settings, with reference standards ranging from histopathology to radiologic or surgical confirmation. Furthermore, the diagnostic thresholds and performance metrics had not been standardized across modalities. Rather than enforcing artificial congruence, we have also presented the metrics as reported in their respective contexts to uphold methodological integrity. This diversity highlights a significant gap in the field, specifically the urgent need for standardized endpoints, harmonized protocols, and uniform reporting criteria to facilitate more meaningful cross-comparison and meta-analysis in future research.

## 3. Overview of Imaging Techniques

This section provides a concise overview of each imaging technology employed in the detection of BC. Every imaging modality possesses its history, principles, advantages, limitations, and uses. In consolidating the results across modalities, prioritized per-patient metrics, including sensitivity, specificity, re-excision rates, and time-to-diagnosis, were selected as they represent the most clinically relevant indicators of diagnostic efficacy. In clinical applications, results have been aggregated and compared whenever feasible. However, the majority of the included studies reported outcomes at alternative levels of analysis, such as per-lesion, per-patch, or region-of-interest (ROI). This data has not been excluded, as it often provides the sole evidence of new modalities, but has specified each measure according to the level of analysis on which it is based. This ensures that the results of the exploratory presentations are displayed in an unconfounded manner, independent of per-patient clinical outcomes. The heterogeneity in reporting is explicitly acknowledged as a limitation of the current body of evidence. This stratified presentation of results aims to balance methodological rigor and completeness, providing the reader with a clear understanding of the clinical applicability and the developmental stage of each modality at present. This underscores the necessity for future research to adopt standardized endpoints on a per-patient basis and consistent reporting practices, enabling meaningful comparisons across studies and bolstering confidence in clinical assertions.

### 3.1. OCT

OCT was initially developed in 1990 and disseminated by researchers at the Massachusetts Institute of Technology in Cambridge, Massachusetts, USA. It was originally employed in ophthalmology for both scientific and clinical purposes due to its applicability in various medical contexts [28]. OCT generates high-resolution cross-sectional two-dimensional pictures of interior tissue microstructures. It was implemented in clinical practice and commercialized in 1996 [29].

**Figure 2:**
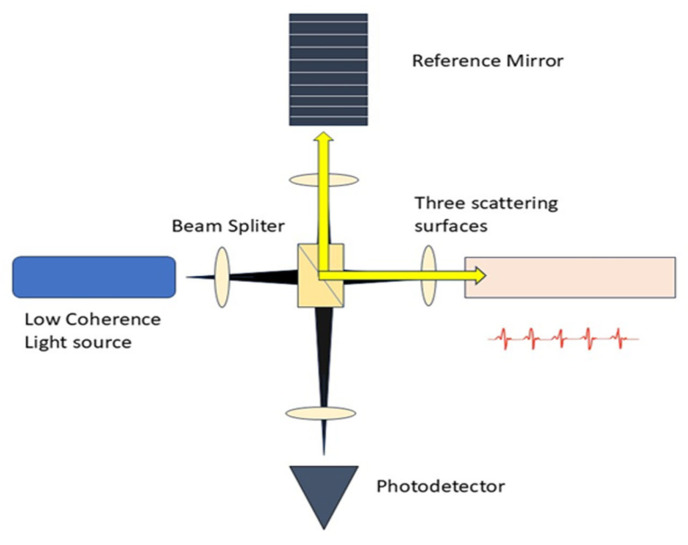
Schematic Diagram of OCT. The setup consists of a low-coherence light source, a beam splitter, reference mirror, and scattering surfaces.

The core concept of OCT involves utilizing low-coherence optical interferometry to detect near-infrared light that is scattered back, thereby reconstructing the depth profile of biological tissue samples. The initial low resolution of OCT devices has been substantially enhanced, allowing for the discernment of smaller and more subtle changes in image quality [30]. It gathers the backscattered and back-reflected light signals from weakly uniform waves that impact upon various tissue depths, as seen in Figure 2. A substantial amount of information is produced through horizontal scanning, subsequently resulting in the creation of a cross-sectional image. The 3D stereoscopic image of the material is ultimately generated using longitudinal scanning [31].

It can conduct real-time, non-invasive, and non-contact measurements in reflection to facilitate three-dimensional sample viewing. OCT necessitates rapid speed or a variable standoff distance between the objective and the sample, particularly when the sample topology is irregular. Light penetration constitutes a significant challenge in OCT [32,33]. OCT is extensively utilized in both scientific and clinical settings, including ophthalmology, dermatology, and gastrointestinal, among others [34]. It possesses a distinctive benefit in lumpectomy, demonstrating that OCT can be adaptively employed in intraoperative clinical practice.

### 3.2. RS

The Raman effect was discovered in 1928 by Chandrasekhara Venkata Raman and Kariamanickham Srinivasa Krishnan. The integration of an optical microscope with a Raman spectrometer in the 1970s utilized laser excitation, enabling the recording of spectra from laboratory specimens for the first time [35]. RS gained prominence in the late 1980s due to advancements in the creation and commercial accessibility of Raman microprobes [36].

**Figure 3:**
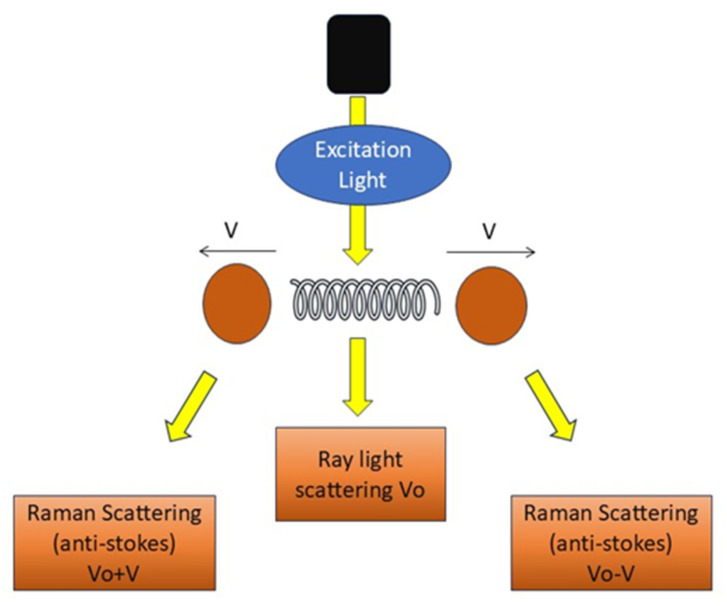
Principle of RS. Schematic representation of RS showing excitation light interacting with vibrating molecules.

RS employs light effects that directly mirror the structures and chemical states of molecules within a sample, as depicted in Figure 3, facilitating the direct viewing of the chemical responses of biomolecules in living cells and tissues [37]. It offers label-free and comprehensive quantitative data of biological material in situ without disturbance. It generates substantial information with excellent precision. Histochemical approaches employed to advance biological sciences are surpassed by RS [38].

RS demonstrates a significant ability to enhance the precision of cancer diagnosis and surgical interventions [39]. RS offers the benefits of being non-destructive, enabling standoff detection, facilitating optical excitation, and possessing potential for portability [40]. Spectral aberrations or elevated fluorescence hinder accurate identification [41]. It possesses two critical deficiencies: limited repeatability and diminished signal intensity [42]. Raman signal-reporting labels may occasionally yield false-positive outcomes [43]. RS applications exhibit great accuracy in the detection of breast, lung, and various other cancers [44]. RS is employed in interdisciplinary applications, such as integrating optics, computer, and microbiology for imaging complicated samples in three dimensions [45]. RS fingerprints are utilized to non-invasively distinguish organoid phenotypes in both fixed and living salivary gland organoids [46].

### 3.3. PAI

The origin of photoacoustic imaging, also known as optoacoustic imaging, traces back to Bell’s serendipitous observation in 1880 that fragmented sunlight striking a solar cell produced audible sound. Since the 1980s, numerous advancements in light delivery technology have enhanced PAI [47].

**Figure 4:**
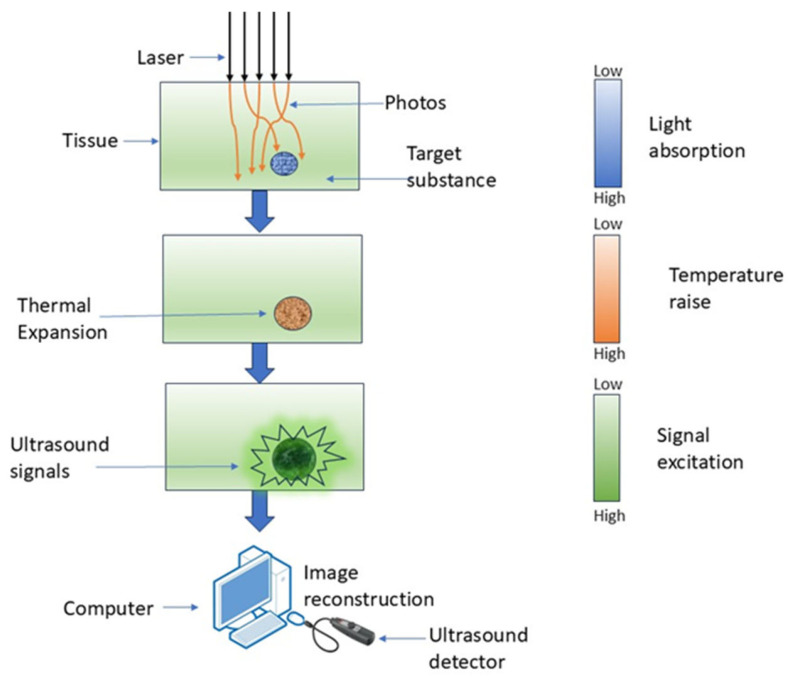
Working of PAI, where laser light is absorbed by tissue, causing localized thermal expansion and generation of ultrasound signals.

PAI is a hybrid imaging technique wherein optical excitation induces thermal effects in tissue, leading to elastic expansion and the production of an acoustic signal [48]. It employs laser-induced photoacoustic signals to produce highly detailed pictures by detecting the acoustic waves generated from the absorption of pulsed laser light by tissue chromophores [49]. PAI non-invasively retrieves functional tissue parameters at depths of several centimeters using the photoacoustic effect, as depicted in Figure 4 [50].

PAI has numerous benefits in high-resolution brain imaging. It has the combined advantages of enhanced optical contrast and higher ultrasound penetration resolution, while also facilitating the quantitative assessment of hemodynamic parameters such as oxygen, hemoglobin, and water concentration using multi-wavelength photoacoustic data [51]. A trade-off exists between imaging resolution and penetration depth in PAI [52]. The bandwidth of the observed photoacoustic waves is constrained [53]. PAI can be utilized in superficial tumors to enhance radiation dose administration, patient classification, therapeutic response, and monitoring of radiation-related adverse effects [54].

### 3.4. HSI

In 1983, the NASA Jet Propulsion Laboratory captured the first hyperspectral image from the world’s first aerosol imaging spectrometer [55]. In 1997, HSI gained prominence in clinical research when Freeman et al. employed it to enhance physicians’ capacity to identify unknown issues in clinical methodologies by providing a perspective beyond the naked eye [56]. In 2006, HSI facilitated in vivo observation of cutaneous edema by enabling the visualization of the water percentage in each pixel, together with oxyhemoglobin and deoxyhemoglobin levels [57]. Brain cancer was identified utilizing HSI in 2018, yielding effective results in the detection of high-grade tumors [58].

**Figure 5:**
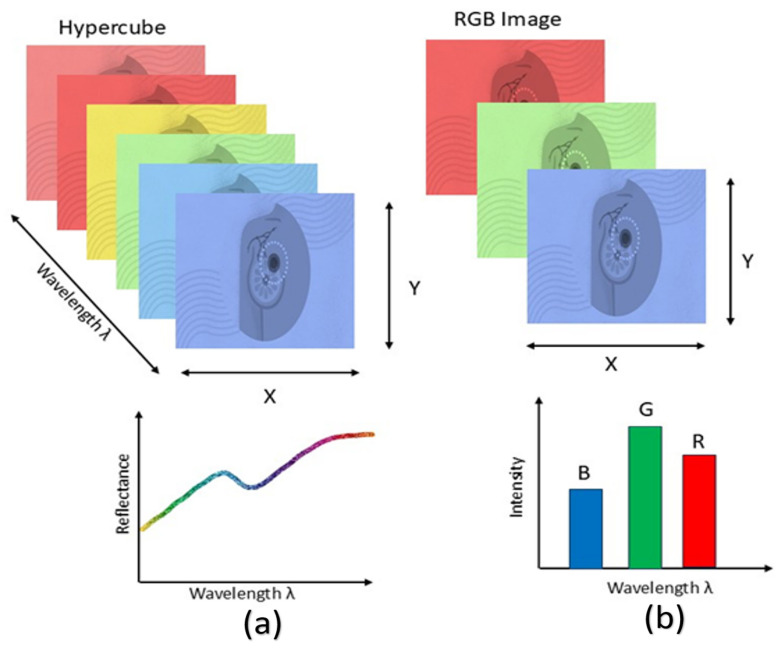
Comparison of HSI and RGB imaging. HSI (**a**) captures information across a wide range of wavelengths, forming a hypercube that provides detailed spectral signatures for each pixel. In contrast, conventional RGB imaging (**b**) captures only three broad wavelength bands, limiting the amount of spectral information available.

When light in the near-infrared wavelength range interacts with biological tissue, it scatters due to tissue heterogeneity and is absorbed by constituents such as hemoglobin, melanin, and water. The fluctuations in fluorescence, tissue absorption, and scattering characteristics during disease alter the spectrum data captured by the device, which conveys pathological information [59]. A 3D hyperspectral cube can be constructed from the generated image data, incorporating spatial information in two dimensions and spectral information in one dimension. The reflectance is related to the absorption and scattering characteristics of cancerous tissue [60]. Figure 5 illustrates the comparison between HSI and RGB imaging.

It is a non-invasive imaging method in comparison to alternatives. Utilizing a wideband light source to assess optical tissue qualities across a broad spectrum of electromagnetic bands [61]. HSI cameras have high adaptability and compatibility with current medical devices, including endoscopes, otoscopes, and laparoscopes [62]. A broad spectral range encompassing the visible spectrum, near-infrared, mid-infrared, and far-infrared, with numerous continuous wavebands included. HSI can also discern items that are difficult to distinguish in natural RGB photos [63].

Hyperspectral cameras continue to adhere to passive imaging technologies. These cameras require an external light source to record objects and depict the intensity of the spectrum. The acquired photos are significantly influenced by the ambient lighting conditions. HSI systems are inoperative in dark or low-light conditions [64]. Higher resolutions lead to extended acquisition durations, creating a trade-off between image quality and data acquisition duration [65]. HSI is an effective technique for medicinal applications. Besides cancer diagnosis and neurosurgery, HSI has been utilized in other forms of image-guided surgery, including abdominal surgeries, cholecystectomy, and renal procedures. HSI is utilized in domains such as remote sensing and food quality assessment [66]. Due to its thorough and complete information, it can be widely utilized across multiple sectors, including agricultural monitoring, geological exploration, and environmental surveillance [67].

### 3.5. CESM

CESM was established in 1985 with the advent of digital subtraction angiography for the breast. The objective was to differentiate benign from malignant breast tumors to prevent surgical biopsy [68,69]. The inaugural study on temporal subtraction was published by Jong et al. in 2003, originating from a group in Toronto [70]. In the same year, Lewin et al. discovered CESM utilizing a dual energy technique, which served as an alternative to the temporal technique [71]. In 2011, the FDA authorized CSEM as a medical method for detecting BC [72].

**Figure 6:**
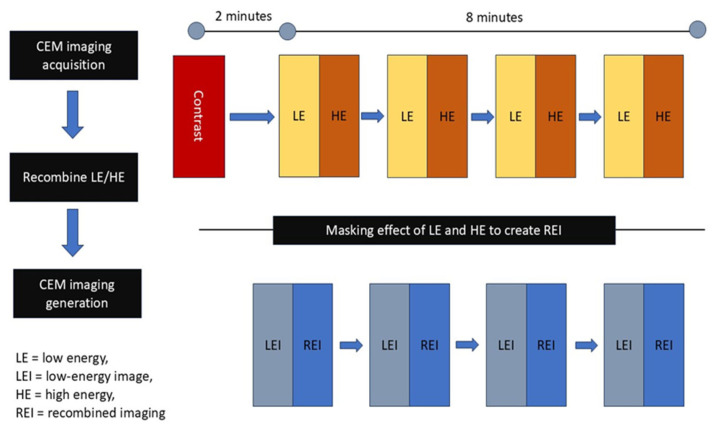
Principle of CESM. The CESM process involves acquisition of low-energy and high-energy images after contrast injection, followed by recombination to generate recombined images.

CSEM relies on the contrast enhancement created by newly developed proliferating tumor vasculature and the elevated permeability in tumor regions [73]. It facilitates both a morphological examination of standard digital mammography and an ongoing assessment of tumor neovascularity [74]. It integrates mammography principles with the administration of an intravenous iodinated contrast medium, enabling a contrast-enhanced graphical assessment of the breast similar to MR imaging; this highlights regions that absorb the contrast medium, indicative of neo-angiogenesis in neoplasms [75]. Figure 6 illustrates the operating concepts of CESM.

CESM exhibits diagnostic sensitivity comparable to breast MRI, and its specificity may be superior in individuals contraindicated for breast MRI. It is as dependable as MRI in assessing the efficacy of Neoadjuvant Chemotherapy [76]. The hormonal condition does not influence CESM. This can offer significant supplementary information about the identification of lesions in individuals exhibiting a pronounced level of background parenchymal enhancement when it is objectively challenging to distinguish a lesion from the non-enhanced background [77]. CESM may be constrained by two factors: firstly, the administration of iodinated contrast medium entails risks of adverse reactions, which may encompass hypersensitivity reactions and contrast-induced nephropathy. Secondly, CESM involves a greater radiation dose compared to full-field digital mammography due to the dual-energy exposure for each projection [78]. CESM demonstrates superior reliability in diagnosing BC in thick breast tissue compared to traditional mammography [79]. CESM is remarkable in high-risk screening and interpretation of ambiguous data, in addition to its function in assessing tumor response to neoadjuvant treatment [80].

### 3.6. MSI

Multispectral satellite remote sensing has a history of nearly 40 years, starting with the launch of the first Landsat satellite in 1972. MSI has been crucial in various satellite and aircraft remote sensing systems. This imaging modality has a long history, having been utilized for over a decade to improve quantitative accuracy, facilitate multicolor immunohistochemistry analysis, and mitigate the effects of contrast-reducing tissue autofluorescence prevalent in formalin-fixed, paraffin-embedded specimens [81,82].

**Figure 7:**
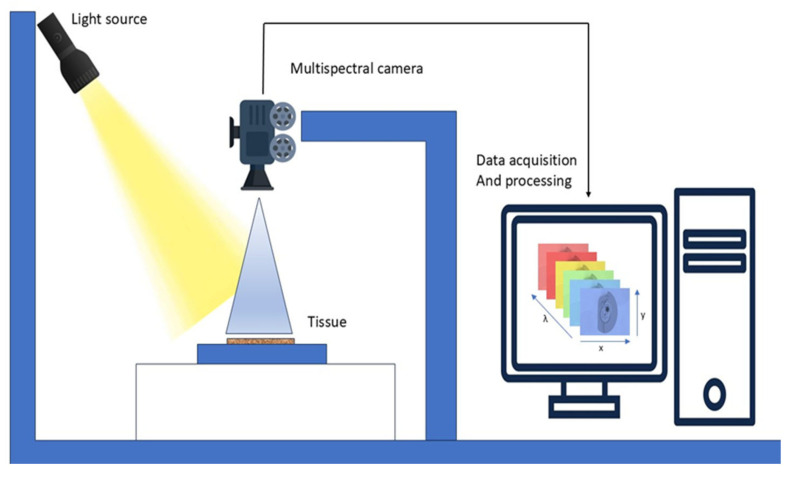
Working of MSI. A light source illuminates the tissue sample, and a multispectral camera captures reflected light across multiple wavelengths.

MSI is also called multiband analysis, involves collecting a set of images from the same area over multiple wavelengths, as illustrated in Figure 7. It utilizes a mix of spectroscopy and imaging techniques to obtain spectral pictures that contain multiple 2-D images. Utilizing the x, y, and lambda axes, MSI constructs a three-dimensional cube. MSI apparatus employs charge-coupled devices, optical filters, and various LEDs over ultraviolet, visible, and infrared spectral ranges to produce reflectance and acquire pictures [83].

MSI integrates enhanced subsets of HSI, making it more efficient [84]. A significant benefit of MSI in cultural heritage is that it does not necessitate the extraction of samples from the object. MSI facilitates the comparison and contrast of various inks and pigments, while also revealing latent features and insights into the current condition of an object [85]. MSI devices have significant potential for the identification and diagnostic evaluation of melanocytic skin lesions by revealing absorption data unseen to the human eye [86].

Current technologies use conventional ML algorithms that encounter numerous obstacles in managing intricate skin lesion circumstances, hence constraining the clinical application of MSI technology [87,88]. Due to the high dimensionality of MSI, processing is slow, and identifying the ideal spectral range is challenging. Various investigations have shown that MSI enhances conventional RGB imaging in the classification of cancer cells [89]. MSI has facilitated significant advancements across many domains like environmental monitoring, astronomy, agricultural sciences, biological imaging, medical diagnostics, food quality control, aerospace, defense, and biomedicine [90]. It is also applicable in forestry and hydrology [91].

## 4. Reported Studies on Imaging Techniques

This section presents many case studies conducted by researchers on distinct imaging techniques, illustrating the methodologies employed, the datasets utilized, and the outcomes achieved in BC detection. It might be tempting to compare performance parameters between different modalities, but this wouldn’t be a good idea because the methods used in each study are different. Presenting results in a synthesized format will provide a comprehensive overview of the existing evidence while preserving the methodological framework of the specific research. The underlying studies are highly heterogeneous, differing in patient cohorts, study design, imaging context (in vivo vs. ex vivo), reference standards (histopathology, radiology, or surgical confirmation), and even the unit of analysis (per-patient, per-lesion, or per-patch). Attempting to normalize these outcomes across such varied conditions would risk misrepresenting the actual performance of the techniques and creating misleading equivalence. Instead, we chose to present the reported metrics in aggregate to provide a broad synthesis of the evidence base, while maintaining the methodological context of each study. This approach highlights the promise of emerging imaging methods while also drawing attention to the current lack of harmonized reporting standards. Future studies should adopt standardized endpoints, consistent reference standards, and multicenter validation to enable meaningful cross-modality comparisons and meta-analysis.

### 4.1. OCT

In the past decade, there have been significant advancements in the application of OCT imaging techniques for the identification of BC. The following case studies demonstrate the clinical and experimental applications of OCT and various techniques, including FF-OCT, CP-OCT, and PS-OCT. These include DL classification models, intraoperative margin intervals, and the integration of imaging and histology, as outlined in Table 1.

The research conducted by Dhiman et al. used OCT images from 48 patients aged 35 to 60 years, featuring a mixture of healthy and malignant breast tissues, collected from the All-India Institute of Medical Sciences in New Delhi. The ensemble model is constructed by ranking and selecting classifiers using the Technique for Order of Preference by Similarity to Ideal Solution, with optimal weights obtained by the Crow Search Algorithm. The results demonstrate that the suggested ensemble classifier exhibited superior performance metrics, with precision, recall, accuracy, F1-score, Kappa, and MCC values of 92.1%, 92.1%, 92.3%, 0.921, 0.846, and 0.846, respectively [92].

Zhang et al. researched 224 D-FFOCT slides of breast tissues derived from 13,497 patches belonging to 129 patients. The Swin Transformer DL model was employed to differentiate between normal and cancerous tissues. The model demonstrated a diagnostic accuracy of 97.62%, a sensitivity of 96.88%, and a specificity of 100%. The intraoperative evaluation technique has been improved, allowing tissue sample assessment to be done in 3 min, markedly less than traditional histopathological approaches [93].

Sanderson et al. utilized data from 16 patients who underwent breast-conserving surgery at Fiona Stanley Hospital in Western Australia, examining 139 in vivo OCT scans and correlating them with ex vivo histology from cavity shavings obtained during the procedure. The spectral domain OCT system was connected to a portable OCT probe. This approach achieved a co-registration rate of 78%, with 109 out of 139 in vivo OCT scans successfully matched to corresponding ex vivo histology [94].

Yang et al. investigated the application of FF-OCT and Dynamic Cell Imaging as non-destructive optical imaging modalities that can be rapidly implemented intraoperatively for the diagnosis of BC surgery. Analysis of a data set of 314 tissue specimens, including 173 breast biopsies and 141 lymph nodes, collected from 158 patients. The research conducted demonstrated the potential for diagnostic efficacy. DCI exhibited superior sensitivity and specificity, reaching 88.6% and 95.1%, respectively, compared to FF-OCT, which demonstrated sensitivity and specificity of 85.6% and 85.4% in breast tissue evaluation [95].

Simon et al. investigated the application of Dynamic Full-Field OCT for the rapid treatment of BC in a clinical setting, utilizing a dataset including 217 samples from 152 patients, which included 144 breast lesions and 61 lymph nodes. This imaging technique combines high-resolution FF-OCT with DCI to provide structural and metabolic contrast without the need for staining or tissue preparation. The outcomes indicated a sensitivity of 77%, specificity of 64%, positive predictive value of 74%, and negative predictive value of 75% [96].

Gubarkova et al. used the cross-polarization OCT technique for imaging intraoperative ex vivo human BC specimens and conducted a qualitative and quantitative evaluation of 3D CP-OCT data through a depth-resolved methodology for estimating the attenuation coefficient and analyzing cross-polarization channels. A study was conducted on 68 excised human breast specimens, comprising both tumorous and adjacent non-tumorous tissues. The research indicates a diagnosis accuracy ranging from 91% to 99%, sensitivity from 96% to 98%, and specificity from 87% to 99% [97].

Faragalla et al. examined the concept of OCT on preserved and unprocessed breast tissue specimens. A total of 175 tissue samples from 40 breast specimens were scanned using a spectral-domain OCT device. This approach identified that 30% of residual malignancy and invasive carcinoma samples could be differentiated from normal fibroglandular tissue based on heterogeneous texture, uneven margins, and diminished penetration depth. The findings supported the efficacy of OCT in producing an accurate image in BC [98].

Levy et al. conducted a study utilizing a substantial cohort of 585 Wide Field OCT margin scans from 151 subjects, employing this data for training and validation, alongside a distinct and independent test set including 155 margin scans from 29 patients with pathology-verified results. The researchers utilized a WF-OCT equipped with a compact Convolutional Neural Network (CNN) model derived from the VGG architecture, tailored for real-time, on-device margin diagnosis during BC. The CNN model demonstrated commendable performance, with an AUROC of 0.976, a sensitivity of 93% and a specificity of 98%. It identified 96.8% of pathology-positive margins, suggesting its efficacy in minimizing reoperations and providing intraoperative decision support [99].

This study by Sun et al. explored the use of paired OCT intensity images and polarization-sensitive OCT images from various human breast tissues to train a DL model. A total of 22,072 PS-OCT images were accurately aligned with the actual images, yielding SSIM scores of 0.8531 for degree of polarization uniformity (DOPU) and 0.6659 for phase retardation. The synthetic PS-OCT attained area under the curve (AUC) values of 0.979 for DOPU and 0.952 for phase retardation in cancer and normal classification, surpassing the natural PS-OCT, which recorded AUC values of 0.975 for DOPU and 0.956 for phase retardation [100].

Basu et al. proposed an advanced full-field polarization-sensitive OCT ensemble model for breast classification, utilizing 220 samples scanned via FF-PS-OCT simulation to extract phase information. The multi-level ensemble classifier achieved a precision of 94.8%, a recall of 92.5%, an F-score of 93.7%, and a Matthews correlation coefficient (MCC) of 82.3% on the testing dataset [101].

### 4.2. RS

**Figure 8:**
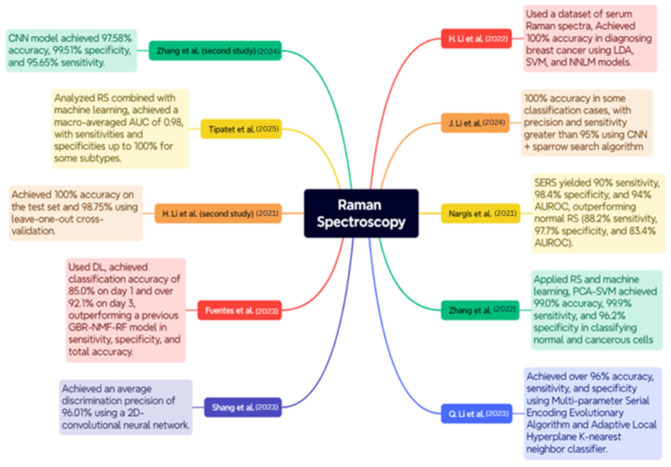
Case Studies on RS for BC Diagnosis [102,103,104,105,106,107,108,109,110,111].

RS is a delicate, non-invasive optical instrument utilized for the diagnosis and classification of BC, as well as for conducting molecular analyses of the disease. The case studies below encompass a wide range of RS applications, including serum and tissue analysis, polarization, and surface enhancement, integrated with ML and DL models to enhance diagnostic accuracy. An overview of the case studies is depicted in Figure 8.

The study conducted by H. Li et al. utilized a dataset consisting of blood Raman spectra from 171 patients diagnosed with invasive ductal carcinoma and 100 healthy participants. The pre-processing stages included baseline correction by airPLS and a smoothing procedure utilizing Savitzky–Golay. The results demonstrated exceptional classification accuracy for models such as LDA, SVM, and NNLM, achieving 100% accuracy in BC diagnosis [102].

The research conducted by J. Li et al. employed many datasets generated by a Monte Carlo sampling method to enhance the model’s validity. A combination of Random Search and CNNs optimized by the Sparrow Search Algorithm. The results indicated that the CNN achieved a 100% accuracy rate in certain classification scenarios, with precision and sensitivity above 95%, hence demonstrating enhanced diagnostic capabilities and effective feature visualization with Grad-CAM [103].

Nargis et al. conducted a study comparing surface-enhanced Raman spectroscopy (SERS) with conventional RS for classifying BC using serum samples from 17 BC patients and 12 healthy individuals. The results indicated that SERS outperformed conventional RS, achieving 90% sensitivity, 98.4% specificity, and 94% AUROC, in contrast to 88.2%, 97.7%, and 83.4% for sensitivity, specificity, and AUROC, respectively, in conventional RS. The findings can corroborate SERS due to its non-invasive ability to differentiate between BC stages and healthy samples in blood serum [104].

Zhang et al. and collaborators employed RS and ML to differentiate between BC subtypes utilizing six cultured cell lines of both malignant and normal breast cells: SUM149, MDA-MB-231, MCF-7, ZR-75-1, BT474, and MCF-10A; approximately 4500 Raman spectra were measured in total. The methodologies employed were Principal Component Analysis (PCA), Discriminant Function Analysis, and Support Vector Machine (SVM) analysis. The PCA-SVM achieved 99.0% accuracy, 99.9% sensitivity, and 96.2% specificity in distinguishing between normal and malignant cells, whereas the classification of BC subtype reached 93.9% accuracy [105].

Q. Li et al. employed a feature fusion methodology for feature identification with remote sensing, incorporating adaptive hyperparameter optimization as a binary classification instrument. The dataset samples were obtained from 16 patients at Peking University. The experiment employed the Multi-parameter Serial Encoding Evolutionary Algorithm along with the Adaptive Local Hyperplane K-nearest neighbor classifier. The model exhibited accuracy, sensitivity, and specificity of 96%, surpassing ALHK with hyperparameter tuning, which is a manual procedure [106].

Shang et al. employed polarized RS to identify structural and compositional changes in breast tissue associated with cancer. The dataset comprised breast tissue samples from patients at Jiangsu Cancer Hospital, collected with ethical approval and informed agreement. The study involved analyzing Raman spectra at various polarization angles to obtain structural information. The training was conducted on this spectral data utilizing a 2D-CNN. The average discrimination precision achieved was 96.01%, beating traditional methods such as KNN and 1D-CNN [107].

Fuentes et al. employed DL techniques based on remote sensing to identify post-irradiation biochemical changes in tumor tissue. A total of 3054 Raman spectra were acquired on day 1 and 6708 Raman spectra on day 3 following the irradiation of breast tumor xenografts. This method employed Random Sampling and CNNs to train and identify irradiated and non-irradiated data. The CNN achieved a classification accuracy of 85.0% on day one, over 92.1% on day three, but exhibited poor performance under leave-one-out validation schemes. CNN demonstrated superior sensitivity, specificity, and overall accuracy compared to a previous GBR-NMF-RF model, establishing it as an excellent choice for early, label-free monitoring of tumor response to radiation [108].

The research conducted by H. Li et al. focused on employing RS for the diagnosis of BC using frozen tissue sections from 22 individuals. The dataset included samples of healthy control, solid papillary cancer, mucinous carcinoma, ductal carcinoma in situ, and invasive ductal carcinoma. Raman spectra were acquired using a confocal Raman microscope with a 633 nm HeNe laser. The researchers employed Principal Component Analysis and Linear Discriminant Analysis to identify the tissue type. The classification model achieved a flawless score of 100% on the test set and 98.75% using leave-one-out cross-validation. These findings demonstrate the efficacy of employing multivariate analysis in conjunction with RS to reliably diagnose BC without the imposition of labels [109].

Tipatet et al. conducted an analysis of the combination of RS and ML for the diagnosis and classification of BC subtypes. The dataset comprises blood plasma samples from 12 BC patients and 12 healthy volunteers sourced from the Biobank and BC Tissue Bank. The study successfully categorized the four major types of BC: Luminal A, Luminal B, HER2-positive, and Triple Negative, achieving a high level of accuracy in classification. The model achieved a macro-averaged AUC of 0.98, with sensitivity and specificity of 90% and 95% for certain subtypes, indicating that liquid biopsy and RS may be highly applicable for non-invasively subtyping early BC [110].

Zhang et al. employed RS in combination with ML to differentiate between normal and late-stage early malignant breast tissues in a mouse model. A dataset comprising images of 20 female mice implanted with 4T1 BC cells, which simulate human stage IV BC, was utilized. A 785 nm laser was employed for Raman measurements, which were analyzed using Random Forest, SVM, and CNN methodologies. The CNN model exhibited superior performance in classification, achieving accuracy, specificity, and sensitivity rates of 97.58%, 99.51%, and 95.65%, respectively [111].

### 4.3. PAI

PAI has developed into a hybrid, non-invasive imaging approach that delivers essential structural and functional information regarding BC tissue by integrating optical and ultrasonic imaging modalities. The following case studies outline the clinical and experimental applications of PAI in BC detection, subtype differentiation, and tumor evaluation, with major findings summarized in Table 2.

A study by Tong et al. used a panoramic photoacoustic computed tomography system to clinically evaluate breast tissue in 39 patients. The optimum classifier achieved an area under the Receiver Operating Characteristic curve (AUC-ROC) of 0.89 for differentiating between normal and diseased tissue, comparable to conventional imaging [112].

G. Li et al. investigated preoperative dual-modal photoacoustic ultrasound imaging for early-stage BC involving 324 patients. The model comprising three independent clinical factors achieved an area under the curve of 0.775 in the training cohort and 0.783 in the test cohort. The comparison indicated that the nomogram exhibited optimal performance, achieving an AUC of 0.906 on the training set and 0.868 on the testing set [113].

Huang et al. introduced a PA radiomics technique to categorize breast nodules among 317 patients with lesions classified as BI-RADS 3-5. Radiomic characteristics were combined with clinical factors by univariate and multivariate logistic regression analysis. The optimal model was MODC, which incorporated clinicopathological, ultrasonography, and SO2 values from PAI, with an AUC ranging from 0.815 to 0.950 and a 95% confidence interval [114].

Li et al. conducted in vivo multispectral photoacoustic imaging to distinguish BC molecular subtypes in mice. Fifty xenografts were acquired by implanting human BC cells into the mammary glands of nude mice. The research utilized each tumor to acquire PA spectra over a range of wavelengths and employed partial least squares discriminant analysis. The Murine model provided significant molecular insights into contemporary approaches. The algorithm demonstrated an accuracy of 72%, with a sensitivity of 66% and a specificity of 78% when utilizing the ideal spectra, surpassing the full range spectra by 6%, 4%, and 8%, respectively [115].

Rodrigues et al. designed a sterile photoacoustic sensor to visualize the in vivo progression of breast tumors and implemented an ML technique for cancer diagnosis, measuring PA spectra at various time points and doing feature extraction. The breast tumor xenografts were produced from five athymic nude mice. A classifier utilizing SVM was developed to differentiate each stage following a tumor from a baseline. SVM-RBF, SVM-Polynomial, and SVM-Linear exhibit accuracies of 95.2%, 99.5%, and 80.3%, respectively [116].

Huang et al. conducted a study utilizing photoacoustic radiomics to predict the Ki-67 proliferation index in 359 BC patients. Multivariate logistic regression was employed to obtain significant clinical variables. The system developed a nomogram for high-Ki-67 status utilizing tumor PA image features, a 6 mm peritumoral margin, and clinical data, achieving an AUC of 0.899 on the test set [117].

Guoqiu Li et al. analyzed the characteristics of deep networks using PA data for breast tumor diagnosis in a patient population of 334, comparing CNNs based on ResNet50 with and without an attention mechanism. The PA input model combined with attention screening achieved the optimal performance, evidenced by a training AUC of 0.917 and a testing AUC of 0.870. The sensitivity, specificity, and accuracy were 78.6%, 87.2%, and 83.6%, respectively, in the test set [118].

Zhang et al. observed a novel approach of multispectral photoacoustic imaging to differentiate between healthy and malignant breast tissues. Formalin-fixed paraffin-embedded blocks of both healthy and malignant human breast tissue were acquired from PrecisionMed, Inc. (Carlsbad, CA, USA). The model employed spectral analysis, incorporating PCA and cosine similarity, utilizing formalin-fixed paraffin-embedded tissue samples from healthy and invasive ductal carcinoma breast tissue. The imaging device detected a wavelength range of 680 nm to 2000 nm to identify tissue-specific absorption characteristics. The mean correlation between healthy tissue and cancer tissue was determined to be between 0.801 and 0.967, and between 0.762 and 0.954, respectively [119].

The study led by Wang et al. examines the application of PAI in evaluating breast intraductal lesions, utilizing a dataset of 45 patients, of whom two were inadequately imaged, twenty-six exhibited non-intraductal malignancies, and seventeen presented with breast intraductal neoplasms, breast fibromas, or breast adenosis. At a PA score threshold of 2.5, the method demonstrated a sensitivity of 90% and a specificity of 87.5% in differentiating intraductal lesions from benign ones. The results suggest that the PAI is a highly promising non-invasive technique for the accurate diagnosis and BI-RADS classification of breast intraductal lesions [120].

Using PAI and radiomics, Guoqiu Li et al. devised a new approach for BC diagnosis to ascertain the pathophysiology of worrisome lesions categorized as BI-RADS 4-5. The research team utilized data from 119 female patients, along with pertinent ultrasound and PAI data. The authors conducted radiomics on PAI pictures, extracting 1125 features, which were subsequently reduced to six significant features by LASSO regression. The average AUC of this composite model was 0.925 during training and 0.926 during testing, far surpassing that of either BI-RADS or PAI radiomics individually [121].

### 4.4. HSI

**Figure 9:**
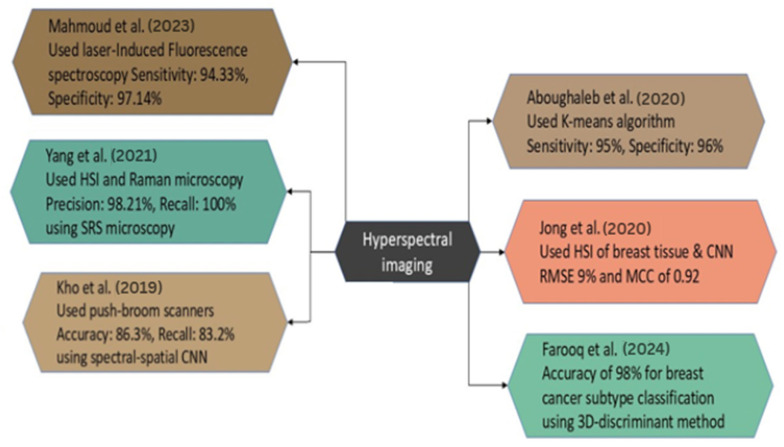
Case Studies on HSI for BC Diagnosis [122,123,124,125,127,128].

HSI represents a novel methodology for the identification of BC. HSI effectively distinguishes between normal and malignant cancerous regions by analyzing the spectrum specific to each tissue type. Recent case studies have employed HSI alongside image processing and ML methods, achieving great accuracy, sensitivity, and reliability. The research examined is illustrated in Figure 9 to offer a comparative analysis of methodologies and outcomes.

Aboughaleb et al. conducted a study focused on ex vivo BC detection with hyperspectral imaging. The scientists analyzed resected breast tissue samples from 10 patients diagnosed with BC, utilizing ethical preparation and imaging with a custom-built hyperspectral camera system across a spectral range of 420–620 nm. The methodology employed in HSI involved the acquisition of hyperspectral data with advanced image processing techniques, including normalization, noise reduction through a moving average filter, and spectral grouping utilizing the K-means algorithm. This facilitated the classification of tissues by distinguishing between normal and malignant tissue signals. The results demonstrated high efficacy, with a sensitivity of 95% and a specificity of 96% in detecting tumor margins [122].

Jong et al. analyzed HSI data acquired from breast tissue slices and lumpectomy specimens from female patients at the Netherlands Cancer Institute—Antoni van Leeuwenhoek Hospital. The authors presented domain adaptation approaches and the spectral and spectral-spatial CNN models to categorize tissue types and evaluate tumor margins. To improve classification accuracy across datasets, a domain adaptation loss function was employed in the methodology. The findings indicated that the models predicted tissue percentages with a root mean square error (RMSE) of approximately 9% and an MCC of 0.92, suggesting a high level of accuracy in distinguishing tumor tissue from healthy tissue during a very brief duration during the surgical procedure [123].

Farooq et al. performed a study on the identification of BC subtypes utilizing HSI samples BT474 and SKBR3, comprising a dataset of 2048 spectra within the fingerprint regime. The primary instrument employed was Fourier-transform infrared spectroscopy, with data pre-processed using a Savitzky–Golay filter and extended multiplicative signal correction. The primary analytical methodology employed was the 3D-discriminant, which significantly outperformed conventional unfolding procedures. The new 3D framework exhibited an accuracy of over 98% in classifying BC subtypes, representing a significant improvement over the 85% accuracy of conventional methods, hence highlighting its potential for personalized treatments. This approach enhanced sensitivity and specificity to 98% and 94%, respectively [124].

Kho et al. generated hyperspectral images of breast tissue, employing two push-broom HSI scanners to capture images in the visible and near-infrared bands. The wavelength range of 450 to 1650 nm yielded optimal classification performance. The authors employ two classification methodologies: a spectral classification algorithm and a deep CNN that integrates spectral and spatial information. The investigation revealed that the highest categorization level was achieved using the complete spectrum of wavelengths and the fiber-optic spectral-spatial method. The recall for U-Net was 86.3%, while LDA exhibited a recall of 83.2%, with both demonstrating identical recall rates for healthy tissues [125].

Halicek et al. examined hyperspectral imaging data from 102 head and neck cancer patients undergoing surgical resection, with 256 measurement sites across tumor mar-gins. To achieve this, the researchers employed convolutional neural networks combined with spectral-spatial feature extraction, enabling automated tissue classification directly from hyperspectral data validated against histopathology. The classification model de-veloped using this approach achieved notable metrics: 91% sensitivity, 83% specificity, an MCC of 0.69, and an AUC of 0.89, demonstrating strong performance in distin-guishing between cancerous and healthy tissue during intraoperative margin assess-ment [126].

The study by Yang et al. focuses on the detailed examination of breast tumor malignancy through the measurement of tissue calcifications using hyperspectral stimulated Raman scattering microscopy. The study utilized specimens from fresh breast biopsies acquired from 23 female patients. In this instance, there were 11 benign cases and 12 malignant cases, respectively. Among 211 patients with identified calcification locations, SRS was employed for imaging. The primary method of analysis employed is hyperspectral stimulated Raman scattering microscopy. The researchers achieved a precision of 98.21% and a recall of 100% in classifying benign and malignant cases. This has been accomplished by optimizing a combination of the chemical and geometrical characteristics of microcalcifications [127].

In the study of Mahmoud et al., an innovative label-free approach for detecting BC was developed utilizing HSI and laser-induced fluorescence spectroscopy. The study included 10 samples of BC, demonstrating that fluorescence distribution serves as an efficient marker for tumor identification. The average sensitivity was 94.33%, and the average specificity was 97.14% across all samples utilizing image segmentation and K-means clustering methods. This non-invasive technology possesses significant potential to improve the accuracy of diagnosis and surgical planning in BC detection [128].

### 4.5. CESM

CESM is an effective imaging technique for detecting BC, as it reveals significant lesions through the use of iodinated contrast chemicals that provide superior image quality. Recent investigations have employed DL, radiomics, and advanced image processing pipelines to improve diagnostic accuracy. The clinical and research characteristics of CESM are delineated in the subsequent case studies and are presented in Table 3.

Jailin et al. used data from 1673 individuals, comprising a total of 7443 contrast-enhanced mammography images collected from various hospitals and imaging centers. The research employs a DL methodology with YOLO for the identification and classification of breast lesions on CESM. The results demonstrate that the optimized model achieved an AUROC of 0.964 in BC classification, successfully recognizing 90% of tumors at a false positive rate of 0.128 per image. The results indicate that the developed AI model surpasses existing studies and closely approximates the accuracy of radiologists in detecting [129].

Bouzarjomehri et al. applied the CDD-CESM dataset comprising 326 patients to develop a DL framework for BC lesion classification. The CESM utilized an attention-based pipeline that enhanced the accuracy of lesion detection and recognition. The classification pipeline demonstrated superior performance, achieving an accuracy of 94.74%, an F1-score of 97.67%, specificity of 93.75%, and sensitivity of 95.45%. Concerning the modality comparison, the model trained on DM images had a little higher accuracy, around 98.85% compared to 97.47% for CM [130].

In a multicenter study conducted by Mao et al., a dataset of preoperative CESM images from 1239 patients, who were pathologically diagnosed, was utilized and divided into training, testing, and validation sets by both internal and external approaches. The primary strategy was employing three prevalent CNN architectures, namely DenseNet121, Xception, and ResNet50, as backbone frameworks, and incorporating the convolutional block attention module to facilitate categorization. The Xception, part of the CBAM family, achieved superior performance, with an AUC-ROC of 0.970, sensitivity of 84.8%, specificity of 100%, and accuracy of 89.1% on the external test set [131].

Chen et al. conducted a study proposing a multiprocess detection and classification system (MDCS) for the detection and automated categorization of breast lesions in a substantial multicenter dataset of 1903 CESM scans from females. In detection tasks, utilizing AUROC as a classification metric, the system achieved average free-response receiver operating characteristic scores of 0.953 and 0.963, with AUC values of 0.909 and 0.912 on pooled external and prospective testing sets, respectively. The MDCS has demonstrated superior diagnostic efficiency, with an average reading time of 5 s, in contrast to the radiologist’s 3.2 min, and it significantly enhances radiologists’ performance when utilized as assistance [132].

Miller et al. conducted retrospective cohort research from 2014 to 2020, reporting on 159 worrisome breast findings sent to the institution for CESM and tissue collection. The study employed logistic regression and penalized linear discriminant analysis to characterize the radially distributed mammographic density and contrast with enhancement. The results indicated that density histograms surpassed a random classifier with an accuracy of 62.37%, while the integration of concatenated density histograms with contrast histograms yielded a notable enhancement, achieving 71.25% accuracy. Furthermore, incorporating both demographic and clinical patient data such as age, race, previous medical history of BC, menopausal state, and breast density into the models yielded an improved AUC-ROC of 0.81 [133].

Moffa et al. compared CESM with traditional tests, specifically digital mammography and breast ultrasonography, for diagnosing women with thick breasts. The study involved 51 patients with 65 breast lesions, collected between March 2021 and February 2022. In the CESM procedure, a non-ionic iodinated contrast agent was intravenously administered via power injection at a rate of 1.5 mL/kg. A mammographic image was subsequently captured, consisting of two acquisitions at low and high energy per view, which were then processed using specialized software to produce recombined images. The data demonstrated that CESM had great diagnostic capability, with a sensitivity of 93.5%, specificity ranging from 79.4% to 82.4%, and a significantly enhanced accuracy of 86.1% to 87.7% [134].

Lin et al. conducted a retrospective study to develop a radiomics nomogram for distinguishing benign from malignant tumors smaller than 1 cm in the breast utilizing CESM. This dataset comprises 139 patients with lesions smaller than 1 cm, including 39 malignant and 100 benign lesions, which are further categorized into training and validation cohorts. Independent predicting factors were determined by ANOVA and multivariate logistic regression. The data revealed that the radiomics nomogram outperformed Rad-score, BI-RADS category, and age, achieving a 0.940 AUC with a 95% confidence interval in the validation cohort, surpassing both Rad-score alone and the clinico-radiological model [135].

Zheng et al. performed a study involving 1912 patients with a discrepant breast lesion identified using CESM. Researchers established a completely automated pipeline utilizing RefineNet and Xception with Pyramid pooling for the segmentation and categorization of breast tissue. It employs a channel fusion technique and pyramid scene parsing networks to utilize the features produced by low-energy and recombined CEM. The system’s performance yielded favorable results, with the Dice similarity coefficients recorded at 0.888 ± 0.101, 0.820 ± 0.148, and 0.837 ± 0.132, respectively. For classification, the AUC values were 0.947, 0.940, and 0.891 in the external and prospective test sets, respectively, indicating a high level of accuracy and potential clinical utility [136].

Gouda et al. conducted a study involving a cohort of women diagnosed with BC who exhibited suspected multifocal or multicentric illness. The dataset comprised 60 patients diagnosed with BC and probable multifocal illness. The authors employed a retrospective research design to examine patients who underwent CESM and contrast-enhanced MRI prior to surgery. The results indicated that CESMs exhibited a sensitivity of 97% and an accuracy of 95%, closely comparable to MRI, which had a sensitivity of 99% and an accuracy of 94%. The specificity of CESM at 67% was significantly greater than that of MRI at 33%, suggesting that CESM may serve as a useful alternative in the preoperative assessment of BC [137].

Research by Song et al. proposed the implementation of hybrid DL to improve BC detection utilizing CESM, which incorporates two imaging modalities: a generative adversarial network (GAN)-based image fusion module and a Res2Net-based classification module. The dataset comprised 760 CESM images from 95 patients aged 21 to 74. The results indicated that the complementary features of both modalities were preserved in the fused images, and the classification model exhibited impressive performance: 94.784% accuracy, 95.016% precision, 95.912% recall, 94.5% specificity, 95.5% F1-score, and 0.947 AUC [138].

Pediconi et al. concentrated on examining the diagnostic efficacy of CESM with high-concentration iodinated contrast medium (HCCM) at 400 mg/mL. Two Italian imaging centers involved 205 patients exposed to CESM, all of whom underwent CEM between March 2021 and February 2022. The findings demonstrated commendable diagnostic accuracy, with sensitivity ranging from 96% to 97%, specificity from 84% to 87.5%, and total accuracy between 93% and 95%. The AUC ranged from 0.90 to 0.92, indicating that CESM’s capability in distinguishing and detecting aggressive breast tumors in conjunction with HCCM is exceptional as well [139].

Sun et al. examined the parameters that may affect the success rate of radiomics models in classifying breast lesions using CESM. The target dataset comprised 157 women and 161 breast lesions, acquired by retrospectively obtained contrast-enhanced mammograms, specifically gathered from November 2018 to February 2020. The radiomic characteristics were obtained using the CESM. The results demonstrated that both models performed effectively. The least absolute shrinkage and selection operator (LASSO) yielded an average AUC of 0.926 ± 0.047, an accuracy of 89.5% ± 6.1%, a sensitivity of 89.1% ± 8.5%, and a specificity of 90.8% ± 9.6%. The Random Forest model yielded an average AUC of 0.915 ± 0.055, an accuracy of 88% ± 6.8%, a sensitivity of 87.8% ± 9.7%, and a specificity of 88.6% ± 10.8% [140].

In this study by Song et al., the researchers created a DL-based classification model called MVMM-Net to enhance the identification of BC using CESM. The dataset comprised 760 CESM images from 95 patients. The procedure comprised three essential steps: preprocessing to integrate the multiview and multimodal capabilities of CESM, deep feature extraction using a Res2Net50 network, and final classification with MVMM-Net. The results indicated a performance accuracy of 96.591%, sensitivity of 96.396%, specificity of 96.350%, precision of 96.833%, F1-score of 0.966, and AUC of 0.966 [141].

### 4.6. MSI

**Figure 10:**
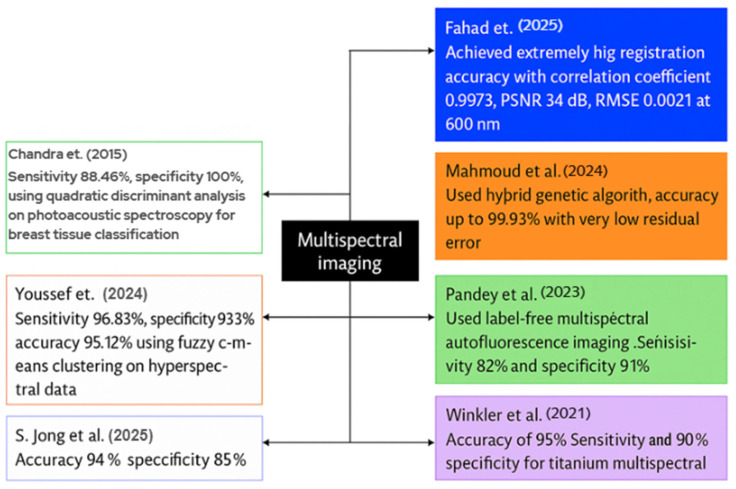
Visual representation of recent MSI case studies in BC detection [142,143,144,145,146,147,148].

MSI enhances BC diagnosis accuracy by providing comprehensive spectral information on tissues. The current scientific evaluations indicate great accuracy rates, contrast, and diagnostic reliability achieved through the integration of imaging, ML, and optimization techniques. This case study will present various MSI techniques employed for BC detection. Figure 10 illustrates a graphic representation of recent MSI studies in the detection of BC.

Fahad et al. introduced a deep-learning system for the registration and enhancement of multispectral breast transillumination images. His team captured transmission images of human participants using a smartphone camera at four LED illumination wavelengths: 600 nm, 620 nm, 670 nm, and 760 nm. The results for 600 nm indicated a correlation coefficient of 0.9973 with an RMSE of 0.0211; for 620 nm, the correlation coefficient was 0.9893 with an RMSE of 0.043; for 670 nm, the correlation coefficient was 0.995 with an RMSE of 0.0248; and for 760 nm, the correlation coefficient was 0.982 with an RMSE of 0.0388. The model employed a hybrid vision-transformer and LSTM network to align and integrate successive multispectral frames [142].

Mahmoud et al. designed a metaheuristic framework to optimize the number of frames required for high contrast in multispectral breast imaging. Two healthy adult female volunteers participated, and the researchers acquired a ten-second video clip utilizing four wavelengths: 600 nm, 620 nm, 670 nm, and 760 nm, across a broad spectrum of LED light. A hybrid genetic algorithm-constriction-factor particle swarm optimization method was employed to align and average the frames at each wavelength. The registration accuracies reached 99.93%, with minimal residual error seen by Mahmoud et al., leading to significant enhancements in image contrast [143].

Pandey et al. examined label-free multispectral autofluorescence imaging for the diagnosis of BC. He, together with colleagues, obtained 172 fresh lumpectomy tissue blocks from 115 patients, utilizing an ex vivo multispectral scanner for autofluorescence analysis. No dyes were introduced; instead, indigenous fluorophores were activated to produce multispectral fluorescence emission data. In validation, the system exhibited a sensitivity of 82%, specificity of 91%, positive predictive value of 84%, and negative predictive value of 89% in detecting malignant and benign zones [144].

Winkler et al. conducted a study including 20 patients; all patients received multispectral MRI immediately before and after the placement of titanium marker clips. The multispectral approach acquired dual-echo data instead of a traditional single-echo image. The objective was to utilize signal variance between the two echo periods to improve the visibility of the metal clips. In the 2-bin MSI model, the sensitivity of clip detection was 90%, the specificity was 100%, and the accuracy was 95% when spectral-image scans were utilized, in contrast to the insufficient detection rate observed with typical single-echo scans [145].

To differentiate between normal and malignant breast histology, S. Jong et al. employed MSI and spectrum unmixing techniques. The group obtained new tissue samples from 189 patients who underwent lumpectomy, utilizing 151 samples for training and 38 for testing purposes. It obtained pure spectral fingerprints of healthy and malignant tissues in the training set using a linear unmixing algorithm. The unmixing characteristics were trained via a K-nearest-neighbor classifier. The findings indicated a sensitivity of 94% and a specificity of 85% in pixel-level tumor detection on the independent test set [146].

Youssef et al. developed a visible-near-infrared MSI set to facilitate the automated identification of BC in tissue samples. The scientists conducted an ex vivo scan of the excised breast specimen across a broad spectrum of wavelengths and employed fuzzy c-means clustering on the hyperspectral cube to delineate the tissue sections. The system demonstrated excellent performance in classifying tumor versus normal tissue regions, with a sensitivity of around 96.83%, a specificity of 93.39%, and an accuracy of 95.12% on the test dataset, including tissue samples [147].

Chandra et al. introduced a photoacoustic spectroscopy method for the label-free identification and categorization of breast cancer tissue. A pulsed laser operating at 281 nm from a Nd:YAG laser system illuminated fresh ex vivo breast tissue samples including 28 normal and 28 malignant specimens. The photoacoustic spectra were captured in the time domain, converted into frequency domain patterns, and examined by quadratic discriminant analysis. The investigation revealed significant variation in tissue types, with the classification model achieving 100% specificity and 88.46% sensitivity within the frequency range of 406.25-625.31 kHz, indicating its potential for clinical breast tumor diagnosis [148].

Khouj et al. reviewed a dataset of hyperspectral pictures of breast tissue specimens from 10 distinct patients, featuring tissue sections on slides that included both hematoxylin and eosin (H&E) stained and unstained samples. To characterize tissues, the researchers employed a snapshot hyperspectral imaging system functioning within the visible spectrum (461–641 nm), enabling the identification of spectral variations between normal and ductal carcinoma in situ (DCIS) tissues. This classification model used the K-means unsupervised clustering algorithm on hyperspectral data cubes, with study indicating that wavelengths approaching 550 nm exhibited the most effective discrimination across tissue types. The classification method attained significant metrics: 85.45% sensitivity, 94.64% specificity, a true negative rate of 95.8%, and a false positive rate of 4.2%, suggesting strong ability in distinguishing between DCIS and healthy tissue for histologic assessment [149].

## 5. Barriers to Clinical Adoption

This section explores the diverse problems encountered by the researchers throughout the investigation. These limitations constitute the primary obstacles to employing these approaches for real-time BC detection.

### 5.1. OCT

#### 5.1.1. Constraints of Physical Imaging Resolution and Penetration

OCT is a helpful tool for examining inside tissues, but it has natural drawbacks. A major concern is its inability to penetrate deeply into the tissue. Gubarkova et al. found that the limited penetration depth of OCT allows scanning to a maximum of approximately 1.5 mm into the tissue. This results in the examination of a limited tissue volume, while the regions of coagulation and hemorrhage also induce alterations in the characteristics of the OCT signal and optical attenuation. Thouvenin et al. stated that the low sensitivity of the faster cameras employed by OCT restricts the penetration depth to 50 μm. This implies that if cancer cells are situated deeper within the tissue, OCT may fail to identify them. The duration needed to obtain information from the sample is greater with FF-OCT due to the constraints of the camera’s frame rate. The integration of FF-OCT with structured illumination microscopy demonstrated inadequate robustness against aberrations and multiple scattering, exhibiting limited penetration into scattering samples beyond 30 to 50 μm [150].

#### 5.1.2. Speed and Real-Time Processing

BC clinical research emphasizes the critical importance of identifying tumor margins to administer appropriate treatment. Given that BC resection can impact women’s appearance and quality of life, it is imperative to provide the most accurate diagnosis. To assist in cancer treatment during surgery, surgeons perform a biopsy to identify the presence of tumor tissues, utilizing Hematoxylin and Eosin (H&E) staining under optical microscopy by a pathologist. The biopsy, which includes resection and H&E staining analysis, may prolong the surgical procedure and complicate the correct identification of tumor margins [151]. During BC surgery, it is essential for surgeons to know immediately whether cancer has been excised. Levy et al. discussed a significant issue when OCT scans a surgical site, such as a lumpectomy margin, resulting in the generation of thousands of minute images. A computer must rapidly verify all of them throughout the ongoing surgery. Conventional DL models such as VGG or ResNet are excessively huge and slow for real-time applications due to their millions of components and parameters. The experiments evaluated 155 margins, corresponding to 1.9 million picture patches, in around 25 min. This results in an average of 10.51 s per surgical margin, with a standard deviation of 6.48 s. If the model is excessively slow, it may postpone surgery and risk the patient’s well-being. Consequently, the speed is crucial in cancer detection models.

#### 5.1.3. Speckle Noise

Speckle is a common phenomenon in coherent imaging systems. When a coherent source and a coherent or noncoherent detector are used to investigate a medium that is rough relative to the wavelength, speckle formation occurs. It resembles a granular structure overlaid on the acquired image, which constitutes a primary constraint in detecting low-contrast lesions. It constitutes a form of noise. Speckle noise can disrupt and influence the assessments of the physician [152]. It is also created by arbitrary interference among numerous reflected photons originating from multiple directions. OCT images are affected by the speckle phenomenon, which serves both as a source of noise and as a carrier of information. It imparts a gritty texture to the OCT pictures. This granular texture diminishes the signal-to-noise ratio (SNR) and reduces the accuracy of interpretation. The primary cause of speckle noise creation is the utilization of spatially coherent light [153]. Speckle noise adversely impacts visual quality and the efficacy of automated analysis, and it hides minor yet critical morphological characteristics [154].

### 5.2. RS

#### 5.2.1. Fails to Extract Discriminative Features

RS is a robust, label-free tool for biochemical study; yet, it often fails to extract discriminative features effectively. Early detection and diagnosis of BC, when tumors are tiny and easily localized, significantly increases the likelihood of survival and facilitates therapy administration. The existing approaches exhibit inadequate detection and diagnosis, with low sensitivity and specificity for the early identification of BC. There is a necessity for the rapid development of dependable systems capable of early disease detection [155]. J. Li et al. observed that current Raman prediction-based models lack visualization and fail to discover critical variables for distinguishing BC subtypes. It highlighted that single-dataset models result in false positives and false negatives, lack an optimization process for model parameters in DL algorithms, and fail to provide good presentation of features. Fuentes et al. have discovered that the Raman spectra of biological samples comprise numerous intricate peaks that simultaneously convey information about multiple biomolecules. Extracting features from extensive remote sensing data is difficult due to the complexity and high dimensionality of the biological spectrum. These limitations show that RS alone is insufficient for high-performance biomedical categorization.

#### 5.2.2. Low Sensitivity of Raman Signals

RS is used to investigate cancer by identifying signals from biological samples; however, these signals frequently lack sufficient strength to yield definitive results. RS possesses significant potential in biomedical and therapeutic applications; nevertheless, these applications are hindered by the weak inherent Raman signals from biological molecules. The extraneous sounds obscure the Raman peaks of interest, resulting in a low SNR. This issue can be resolved by augmenting the excitation laser power and extending the exposure time. This technique is not applicable for measuring unstable materials or for viewing rapidly changing events [156]. Nargis et al. found that the Raman signal is the main limitation of the approach, and the concentration of principal metabolic products in bodily fluids, such as blood serum or plasma, may fall outside the detection threshold of standard Raman scattering. Raman signals must be enhanced to analyze biofluids.

#### 5.2.3. Manual Feature Selection and High Dimensionality

DL is used with remote sensing to identify essential features; nonetheless, issues persist in extracting global sequential features. The Raman spectrum is depicted by Raman intensity across different Raman shifts. Continuity exists in Raman intensities corresponding to adjacent Raman shifts in RS, leading to a certain coherence in the sequence of the Raman spectrum. These generate a global sequence that results in improper feature extraction and subsequent information loss. In DL, the convolutional kernel size is constrained, resulting in the extraction of only partial features [157]. Collaborating with RS for BC detection presents significant challenges due to the vast quantity of intricate data it generates. H. Li et al. discovered that the Raman data contained excessive characteristics, resulting in models that were sluggish and difficult to utilize. The large dimensionality of spectral data will result in longer model operation time. Y. Zhang et al. faced difficulties with the extensive information within the data and observed that conventional methods for analyzing Raman spectra primarily depend on feature selection and linear statistical models, which may fail to encapsulate the high-dimensional, nonlinear interactions intrinsic to the data. These constraints can influence the precision and reliability of cancer diagnosis via RS. It also complicates the effective differentiation between distinct types and stages of cancer. These manual techniques are difficult and open to human error. The complex structure and high dimensionality of Raman data render its proper utilization challenging without considerable time and specialized expertise.

### 5.3. PAI

#### 5.3.1. Incapable of Accurately Identifying Malignant Tumors

Finding BC using PAI remains challenging. Lin et al. used a specialized imaging technique known as SBH-PACT to see if BC had been completely removed after chemotherapy treatment. However, the PACT studies were unable to detect subtle angiographic structures, such as individual blood arteries, due to system constraints, including inadequate spatial sampling rate and restricted detection angle. Angiogenesis measurements have not been used in imaging due to the inability to reliably detect microvascular changes. These compromised the model’s accuracy and reliability. Li et al. had problems with a similar imaging system that used a DL methodology and a ResNet50-based model combined with an attention mechanism to analyze photoacoustic ultrasound images. Categorizing blood oxygen signals into low, medium, and high improves the diagnostic procedure. Reliance on visual interpretation may result in the overlook of subtle textural features, hence affecting the accuracy of cancer diagnosis. Radiologists encounter challenges in accurately detecting and diagnosing malignant tumors in breast lesions using this approach. These findings indicate that PAI continues to exhibit deficiencies and is not consistently capable of accurately detecting BC.

#### 5.3.2. Limited Angular View

In PAI the reconstruction algorithms are only exact if the detection surface or curve surrounds the object. Any deviation from the ideal closed detection geometry results in a limited view or limited-angle problem. This limitation exists and raises concerns regarding the accuracy and stability of the reconstructions. The main problem with PAI in detecting BC is that the tool is unable to examine the breast from all angles. As a result, the images it captures are occasionally fragmented or indistinct. In limited-angle photoacoustic imaging, the absence of data results in artifacts that can be classified as Missing Information outside the detection region, leading to spatially inconsistent blurring of reconstructed images. Points assigned varying weights due to differing detection angles are rebuilt with inaccurate relative amplitudes. Within the detection region, artifacts emerge from redundant directions [158]. Lin et al. faced the same problem; the system has limitations such as insufficient spatial sampling rate and limited detection angle, thus limiting the accuracy and reliability. These studies showed that not being able to see the whole breast is a major issue and can make it harder to find BC early.

#### 5.3.3. Lack of Rigorous Quantitative Comparison Methods Between Volumetric PAI and Histology

Sangha et al. found a key problem: the absence of quantitative methodologies that systematically compare volumetric photoacoustic tomography (PAT) and ultrasound pictures with gold-standard histology, hence constraining clinical validation and translation. Comparing gold-standard histology, clinical imaging, and photoacoustic data presents inherent challenges. The discrepancies in the PAT reports may stem, in part, from inadequate co-registration techniques for precise comparison of histology or gold-standard clinical imaging. This complicates the demonstration of the efficacy of these novel imaging techniques for cancer therapy. The scientists had difficulties matching detailed images from PAT and ultrasound with histology due to challenges in achieving exact alignment and the lack of standardized measurement and comparison methods. Consequently, physicians have not extensively employed these new imaging modalities, as the correlation between the pictures and actual tissue remains uncertain.

### 5.4. HSI

#### 5.4.1. Speed of Data Acquisition

Data acquisition is the process of gathering information from the real world, mostly in numerical form, which is then analyzed and stored. The use of a computer automates the data acquisition process, enabling quicker data collection with fewer errors [159]. One major problem with employing HSI for BC detection is the long time required to obtain high-quality images. This is especially true when examining large cells or big areas of tissue. Farooq et al. were unable to collect the samples due to high resolution and the considerable acquisition time needed to acquire co-added scans, which rendered the collection of several samples during short intervals unfeasible. This is a problem in hospitals where doctors need fast results. Different methods of measurement for large cells require more time to obtain high-quality images, limiting their use in the clinic due to the pace of data collection and the absence of efficient computational protocols. The longer scanning procedure complicates quick decision-making during surgery or diagnoses. Recent research has facilitated the method a bit; however, it still requires several minutes. That is too slow for real surgeries, where time is critical. Both studies show that although HSI gives valuable and comprehensive information, it is still too slow for easy application in hospitals. This issue must be resolved for more frequent utilization.

#### 5.4.2. Obstacles in Direct Clinical Translation and Validation

A major challenge is that the HSI approach works well in lab settings, but it is hard to implement in real surgical environments within hospitals. Kho et al. achieved high sensitivity and specificity, indicating the potential of HSI as a margin evaluation tool to enhance surgical outcomes. Complete tumor excision during breast-conserving surgery can be challenging due to inadequate intraoperative margin assessment techniques. Ortega et al. also said that further research is necessary to ascertain the possible clinical applications of HSI. Jong et al. stated that the current techniques are ineffective in assisting surgeons and admitted that the model fails to accurately predict the interaction between light and inhomogeneous tissue, potentially leading to erroneous values. So as long as these methods undergo careful evaluation in real surgical procedures, hospitals may refrain from their implementation.

#### 5.4.3. High Cost and Lighting Dependence

The complexity, high cost, and large size of HSI equipment constitute a significant obstacle to the general implementation of this technology. HSI additionally generates large and very valuable datasets, which have many features and hinder appropriate interpretation [160]. Data-intensive characteristics and instrumentation challenges of HSI have limited its overall adoption. In instances of insufficient illumination, the SNR of hyperspectral data presents an additional hurdle. The poor SNR results in untrustworthy data, compromising the accuracy of analysis and interpretations. Managing and analyzing the huge data produced by HSI necessitates increased computational resources and skill. The technology’s sensitivity to environmental variations can diminish data accuracy. The HSI technology also shows lower spatial resolution compared to conventional imaging techniques. This impacts the accuracy of data and the use of HSI. HSI faces problems like as multicollinearity and incomplete or noisy data, which hinder product characterization. Enough light is essential for effective HSI operations; inadequate lighting might damage image quality, necessitating increased efforts in data processing. HSI equipment is costly relative to other imaging modalities and detection approaches due to the high expense of the high-definition cameras integrated into HSI systems [161].

### 5.5. CESM

#### 5.5.1. Absence of Automatic Segmentation

Numerous studies found an important problem with finding breast tumors with specialized mammography pictures, as there was no automated method to delineate the tumor regions. In the study by Mao et al., regions of interest for the breast lesions were drawn manually by a senior radiologist. The manual method was applied for image segmentation, resulting in a good DSC. However, the automated approach eliminates human error and participation from image segmentation to the creation of the prediction model, hence improving the repeatability of research and the practicality of the model. Using a fully automated system that works independently of human intervention could improve the consistency and usefulness of the outcomes. No literature was found regarding the automatic segmentation of breast lesions on CESM. The study by Chen et al. encountered the same issue. Image annotation was conducted by two independent radiologists with 10 to 14 years of expertise. A rectangular box was drawn around the breast lesion using ITK-SNAP. In cases of cancer in situ, lesions were hardly observed in RC images because of unclear enhancement. This process is time-consuming, dependent on the radiologist’s assessment, and makes it harder to use more facilities, particularly in complicated scenarios. Both studies agreed that future studies should focus on the development of completely automated systems capable of independently detecting and delineating tumors.

#### 5.5.2. Insufficient Database Size

A major problem in employing advanced imaging techniques for BC detection is the inadequacy of medical pictures. Jailin et al. said that a few research projects using contrast-enhanced mammography were conducted due to the limited availability of extensive picture datasets. Due to the limited sizes of the available databases, most of them have only a few hundred cases, necessitating that researchers manually select and extract Region of interest (ROI) for classification, hence making the process slower and less accurate. Limited datasets hinder the assessment of the efficacy of approaches in real-world scenarios, such as across various hospitals or among diverse patient populations. Zheng et al. found that small-sample and single-center datasets are inadequate for successfully acquiring diverse image features and do not satisfy the criteria for generalized performance evaluation. The method used CESM with DL, which is highly dependent on data; thus, more data from other institutions is required to construct a predictive model that captures the diverse characteristics of breast lesions. Both studies agreed that insufficient high-quality and diverse images may limit the efficacy of imaging approaches to a narrow range of scenarios, thus compromising their reliability in real-world applications.

#### 5.5.3. Increase in Radiation Dose

One major drawback of CESM is that it requires the patient to undergo additional radiation doses compared to conventional breast imaging methods, including routine digital mammography or sonography. While the latest procedures enable physicians to obtain a more comprehensive image of BC, particularly in women with thick breast tissue, there is, regrettably, a trade-off involving a greater radiation dose. A study by Moffa et al. indicated that CSEM may result in an increased radiation dosage compared to digital mammography, varying between 15% and 80%, alongside the utilization of iodinated contrast media and elevated expenditures. Since the radiation dose is elevated, it remains below acceptable limits, and severe adverse responses to the contrast agent are exceedingly rare, approximately 0.002%. Gouda et al. observed that, while CESM is a valuable imaging modality for the evaluation and diagnosis of BC, exhibiting comparable sensitivity and accuracy with increased specificity, it requires a larger radiation dosage. Therefore, clinicians must carefully evaluate whether improved scanning would yield beneficial outcomes alongside any risks related to heightened radiation exposure.

### 5.6. MSI

#### 5.6.1. Human Respiration and Camera Instability

The quality of MSI is affected by several aspects, including intense light absorption, light scattering, camera instability, and the motion of patients. This factor results in noisy images with diminished grayscale resolution and also compromises the quality of the images essential for effective treatment. Mahmoud et al. indicated that factors such as camera jitter and respiratory movements during image acquisition can result in misalignment, hence compromising the quality of the resultant image. Displacement of image frames occurs during sequential capture due to respiratory movements and camera jitter. This gives very blurred and distorted images. Fahad et al. also discovered that the accuracy of results may be undermined by motion induced by human respiration and camera instability occurring during the transition between image frames. The challenge of frame shifting arises from patient motion and camera instability. Failure to address these variables diminishes the accuracy of MSI, necessitating enhancements in noise reduction and the mitigation of patient motion effects.

#### 5.6.2. Spectral and Spatial Resolution

MSI measures spectral data crucial to the absorption and scattering of light. MSI can acquire both spatial and spectral data. Spatial and spectral resolutions vary based on the specific applications. Spectral resolution is defined as the differentiation between two consecutive spectral channels in the sampling process of the electromagnetic spectrum. MSI uses broad bands that may obscure essential characteristics. A narrow spectral resolution facilitates the identification of tiny absorption peaks that cannot be discerned at lower spectral resolutions. The use of low spatial resolution can result in spectral mixing, which refers to the identification of blended spectral signatures from many materials present within a certain pixel. The spatial resolution pertains to the actual pixel dimensions. Low spatial resolution and strong spectral mixing are used only when the application pertains to homogeneous materials or materials that show enough homogeneity for such spatial granularity. Current imaging techniques render it nearly impossible to ascertain the optimal parameters, including wavelength, spectral resolution, and spatial resolution, for each specific application [162].

#### 5.6.3. Issues with Calibration & Standardization

MSI must be accurately calibrated to address the data heterogeneities introduced by the imaging instrumentation. The lack of standardization in current information extraction methods complicates cancer identification. Data analysis is sometimes done to provide insights into data calibration, with the primary objectives being classification and segmentation. Spectral capture equipment is unstandardized, intricate, research-grade, and expensive. The instrumentation significantly changes between different investigations, resulting in ambiguity. The optimal apparatus parameters for HSI histological analysis remain unclear. The hardware is prohibitively expensive, necessitating substantial data storage and imposing exceedingly high processing needs. These systems also face challenges, such as massive data volumes and costly processing demands. A significant difficulty that cannot be ignored is that the data storage size exceeds that of RGB digital pathology, which lacks clear solutions. Instrumentation is costly and lacks standardization, and cost–benefit analyses are also absent in MSI [163].

## 6. Future Directions

This section presents solutions to the restrictions discussed in the previous section. These strategies are effective in addressing the many limitations encountered in imaging techniques for BC detection. This approach provides precise and effective methods to address the limitations of imaging techniques. The primary application of the emerging imaging modalities examined in this paper is for the diagnosis and characterization of primary breast tumors, as well as the intraoperative assessment of the surgical margin. The most advanced methods available right now are OCT, RS, HSI, and MSI. These methods provide detailed information about the tumor’s shape, size, and type of tissue. There are also modalities, particularly PAI and CESM, that show it is possible to check regional lymph nodes because they can show vascular patterns and perfusion features. Nonetheless, the utilization of these modalities for the detection of distant metastases remains in the preclinical or exploratory stage, lacking sufficient evidence to warrant routine clinical implementation. This difference shows that these methods could be used for more than just examining primary tumors, but they have only been proven to work for primary diagnosis and surgical pathfinding. More research is needed to see if they can be used for metastatic disease as well.

### 6.1. OCT

The penetration depth of most of the OCT techniques is limited and constrained by light scattering within tissue. Recent studies offer hardware and optical modifications to improve the shallow penetration of OCTs. One of the most effective strategies is to employ dual-axis OCT, which enables deep tissue imaging through novel off-axis illumination or follows a proper detection configuration. Zhao et al. conducted a study on dual-axis OCT design, achieving enhanced depth penetration. DA-OCT gives a hundredfold improvement in speed compared to its predecessor, multispectral multiple scattering low coherence interferometry, by employing a scanning technique with a microelectromechanical system mirror [164]. Optical clearing chemicals and alternative clearing methodologies can enhance depth penetration. Shariati et al. employed temporal optical clearing, an agent-free technique that reduces both light absorption and scattering by pulse width modulation in the ultra-short pulse regime. Ultrasound optical clearing can enhance light penetration depth by three mechanisms: impacting the tissue surface, creating gas bubbles inside the tissue, and creating a waveguide from the surface to the tissue’s depth. The integration of ultrasonic and agent-based approaches enhances the depth of OCT to 1.3 mm. This provides speedy, efficient, non-destructive, and effective agent-free optical cleaning procedures with deep tissue penetration [165]. Wide-field OCT can enhance depth of penetration by rapidly capturing a larger volume of data. The software organizes these images into a stack for user viewing, offering microscopic vision of cross-sectional areas while enhancing depth up to 2 mm beneath the sample’s surface, with a maximum size of around 9 cm × 9 cm and a resolution of 30 μm [166].

Nowadays, the speed of the model or modality is important due to the necessity for prompt outcomes. OCT is unable to give results promptly; hence, numerous researchers are employing optimized deep neural networks (DNN) for real-time margin assessment, achieving performance markedly superior to existing literature and approaching the proficiency of human experts. Lightweight models, such as CNNs, are utilized instead of hefty ones. Triki et al. developed a DNN for real-time processing, which outperforms the current research and aligns with the ability of human experts. Using the technique of DNN yielded significant results and demonstrated efficacy for small images extracted from patches comprising exclusively cancerous or normal tissue, as identified by pathologists at a university hospital. This method proved computationally viable for intraoperative use, necessitating under 2 s per image for the report [167]. Wang et al. employed DL for rapid and precise intraoperative evaluation of OCT images augmented with polarization-sensitive augmentation. ML and DL can improve the speed and accuracy of image analysis during real-time surgery [168].

To remove the constraints imposed by speckle noise in OCT, Gomez Valverde et al. devised a speckle noise reduction technique that leverages adaptive noise wavelet thresholding in conjunction with a wavelet compounding procedure used on many frames captured from successive places. This method employs a wavelet representation of the speckle statistics derived from a homogeneous sample or the area of the noisy volume. The technique first processes several frames collected at successive places using a multiscale noise-adaptive wavelet filter, followed by a suitably weighted computation for compounding. This enhances speckle noise elimination and augments structural details, resulting in model performance that accurately compares to other state-of-the-art techniques. Ma et al. introduced edge-sensitive conditional GANs that concurrently diminish speckle noise. The edge loss function was introduced, providing effective edge preservation while simultaneously smoothing related areas. Liba et al. demonstrated speckle-modulating OCT, which was solely reliant on light manipulation. It efficiently eliminates the speckle noise originating from a sample. SM-OCT does this by creating and averaging an infinite number of picture scans with uncorrelated speckle patterns while maintaining spatial resolution. This little structure was also revealed [169].

### 6.2. RS

Sometimes, RS fails to extract distinguishing features from complex samples. This is addressed by various methods, such as data-driven feature learning. In 2017, Liu et al. trained a deep CNN to recognize distinguishing features. CNN has been designed to extract features from an input signal at various levels of abstraction. CNN is designed to automatically identify substances according on the Raman spectrum, removing the need for ad hoc preprocessing processes. CNN has shown significant success as an end-to-end learning system, as it automatically processes data and collects features, hence avoiding the need for manual feature engineering. CNN demonstrated good accuracy. Numerous studies suggest that in order to extract all discriminative features and ensure precise feature selection, the model or tool must use different feature extraction methods, such as principal component analysis (PCA) and partial least squares discriminant analysis [170]. Guo et al. used PCA and PLS approaches to identify features indicative of intergroup differences. PCA improves the accuracy of predictions on testing data that significantly differs from the training data, whereas the PLS technique mitigates overfitting, resulting in more stable classifications [171]. Advanced techniques, like as deep networks and optimized feature engineering, enhance the selection of features and the emphasis on intricate minor features within tissues, hence enhancing accuracy and detection or classification rates.

Weak Raman Signals are unable to detect the cancerous tissues, and the signals are too weak to give clear results. Enhancing the Raman signal is important, along with facile surface modification and ML, to tackle the aforementioned challenges. There are various ways to amplify weak signals. Currently, surface-enhanced Raman scattering and nanoparticle substrates are widely used to mitigate weak signals. Three-dimensional ordered nanostructures are used for enhancing the signal. Colloidal plasmonic metals, including silver and gold nanoparticles, are used to enhance Raman intensity by electromagnetic effects. To enhance the efficiency of Raman signals, various nanomaterials with different shapes, such as nanostars, nanorods, and nanocubes with numerous branches, were explored. Semiconductor materials, either organic or inorganic, have inherent Raman signal enhancement by charge transfer mechanisms, finding a new way for SERS. EM derives the enhancing ability from electromagnetic effects on nanostructured metallic surfaces reaching up to factor of 10^4^ to 10^10^ [172]. Lin et al. used a serum-based platform that integrates surface-enhanced RS technology with resampling strategies, DL, feature dimensionality reduction, and interpretability analysis approaches to get sensitive and accurate results. The use of this technique produced good results, with an accuracy of 93.15% [173].

High dimensionality is a major obstacle in RS. Some images may have various features that complicate the detection of malignant tissue. Various dimensionality reduction techniques exist. Principal component analysis is the most common method used. PCA axes can be regarded as a novel set of features that encapsulate the majority of the spectral information and can later be utilized by automated ML models. PCA reduces the number of variants but retains the most important features necessary for cancer detection. Other dimensionality reduction approaches include t-SNE and uniform manifold approximation and projection. This technique identifies and reveals nonlinear manifolds in the projection space through nonlinear transformations. Autoencoders are utilized for dimensionality reduction. It is a variant of DL aimed at extracting latent features from unlabeled datasets. Dimensionality reduction is achieved by employing an encoder to compress the data into a lower-dimensional space known as latent or code space, followed by reconstructing the input to restore it to its original dimensions at the output [174].

### 6.3. PAI

The main limitation of PAI is the inability to accurately detect malignant tumors. It can happen because of the inherent features of the tissues that create contrast in imaging. Various strategies exist to surmount this challenge. Li et al., 2019, found a way to detect malignant tumors. The method developed pH-responsive gold nanoparticles that possess specific targeting capabilities for cancer cells and emit distinct signals in acidic tumor microenvironments. The nanoparticle surface was modified with two thiol conjugate molecules that effectively stabilize it at the pH of blood and normal tissues. The study on the effects of surface conjugate molecule composition was conducted systematically, resulting in a successful creation of a pH-responsive active tumor-targeting nanoprobe, which showed a significantly increased contrast effect for both in vitro and in vivo PAI [175]. Lin et al. developed a single-breath-hold photoacoustic computed tomography that revealed detailed angiographic structures in human breasts. SBH-PACT helps in finding detailed angiographic structures within human breasts. SBH-PACT exhibits profound penetration depth alongside elevated spatial and temporal resolutions. A volumetric image can be obtained by scanning the entire breast in a single breath hold and subsequently rebuilt using 3D back projection, resulting in minimal motion errors caused by breathing. SBH-PACT accurately recognizes tumors by detecting elevated blood vessel densities associated with tumors at high spatial resolution, demonstrating early potential for enhanced sensitivity in radiographically dense breasts [176].

Another disadvantage of PAI is that it allows only a limited angular view of the sampled data. This limitation hinders the recognition of malignant tumors and obstructs the sufficient acquisition of complex tissue structures. To overcome this issue, Sun et al. developed an approach for enabling fast 3D full-view imaging. An array of hemispherical ultrasonic transducers can be integrated with a planar acoustic reflector. It functions as an ultrasonic detection device in photoacoustic computed tomography. The main idea of using this is that a planar acoustic reflector can provide a mirrored virtual transducer array, hence extending the detection range. It can expand the detection view to approximately 3.7 π steradians in the detection configuration. This approach possesses significant potential for both preclinical and clinical research by delivering thorough, precise, and comprehensive images of biological tissues [177]. An acoustic reflector at a 45-degree angle can also be employed to address this problem. The reflector forms a virtual array that is orthogonal to the physical array. The reflector is optically transparent, enabling more versatile light illumination schemes. The utilization of an acoustic reflector successfully doubled the detection coverage of the tissue sample [178].

The lack of rigorous quantitative comparison methods between volumetric PAI and histology presents a significant hurdle for PAI. It also lacks clinical validation and applicability. Dual modality PAT and ultrasound imaging have the potential to increase breast margin characterization by delivering clinically relevant compositional information with high sensitivity, hence improving intraoperative BC diagnosis. Sangha et al. made a study to address this issue and presented a quantitative multimodal workflow utilizing inverted Selective Plane Illumination Microscopy (ISPIM) to enhance image co-registration between volumetric PAT ultrasound datasets and histology in human invasive ductal carcinoma breast tissue samples. ISPIM is an optical imaging technique and a variation in light sheet microscopy that assists in resolving image co-registration challenges for the validation of volumetric label-free imaging techniques. This method enables users to obtain high-resolution volumetric datasets at imaging rates 30 times quicker than tissue cleaning techniques [179].

### 6.4. HSI

High-dimensional data cubes with detailed spatial and spectral resolution experience diminished data acquisition speeds while collecting large datasets. Traditional methods require long capture durations, from several minutes to hours, hence limiting the feasibility of HSI. To overcome this challenge, Holman et al. developed a gridless autonomous adaptive sampling approach. This technique substantially decreases data collection time while increasing sample density in regions with pronounced physicochemical gradients. This gridless adaptive sampling method outperformed standard uniform grid sampling in Fourier Transform infrared spectroscopy studies within a two-component chemical model system. This method is viable, more efficient, and has a greater chance of success [180]. Takizawa et al. faced a similar problem, which was overcome by integrating time-domain HSI and compressed sensing. This approach achieved a tenfold increase in image acquisition rate compared to conventional raster scanning [181]. The technologies mentioned above significantly decrease the data acquisition time for biological tissues.

A significant challenge in the direct clinical translation and validation of HSI is the adaptation of complex optical and computational technologies to the context of real-world clinical practice. Some studies, such as that by S. Jong et al., achieved great accuracy in classifying hyperspectral pictures of lumpectomy, allowing for the imaging and evaluation of the entire resection surface within 10 min. This provided a rapid and non-invasive alternative to conventional margin assessment techniques. This finding provides substantial advancements in real-time intraoperative margin assessment [182].

HSI’s functionality is compromised in dark or low-light environments, hindering effective operation of the system. Prototype devices for active HSI in low-light conditions exist. Tang et al. built prototypes that actively emit various single-wavelength light rays at appropriate frequencies during the imaging process. The method has several advantages, like using controlled lighting, which standardizes the magnitude of the single band for obtaining reflectance information. The device might also concentrate on the specified spectral region by adjusting the number of LEDs. The system is also mechanically easier to manufacture [183]. Stuart et al. introduced a low-cost smartphone-based HSI system that converts a standard smartphone into a visible wavelength hyperspectral sensor for £100. It was the first smartphone capable of collecting hyperspectral data without requiring considerable post-processing. Smartphone camera sensors are capable of capturing images with a high resolution [184].

### 6.5. CESM

Recent developments in DL techniques have emerged for automating the process of breast lesion segmentation in CESM images, reducing the necessity for manual outlines. Khaled et al. released a Categorized Digital Database for Low Energy and Subtracted CESM Images to evaluate support systems. The EfficientNetB0 model was trained to predict the overall diagnosis, which consists of normal, benign, and malignant tumors. GradCAM was used to highlight highlights for the areas of the sample that contribute to the model prediction and performance [185]. Zhu et al. developed a VNet architecture for segmentation, together with the ResNet model for classification, to perform automated analysis in a multicenter study involving 3303 patients with multiparametric MRI. The deep learning CADx model demonstrated potential for the segmentation and classification of breast lesions, exhibiting good generalization abilities and clinical application [186].

To address the constraint of limited database sizes in medical imaging applications such as CESM, researchers developed various new solutions that enhance model performance. Transfer learning is one of the most effective methods to solve this issue. It was implemented to diminish the necessity of the annotation procedure by transferring DL models with information from previous tasks and then fine-tuning them on small datasets. A Deep CNN model was proposed that combined recent advances in the field. The model attained an accuracy of 85.29% when trained from inception and 97.51% with the proposed methodology in BC detection. The proposed technique enhances the performance of both classification scenarios [187]. Advanced data augmentation approach can potentially be an important solution to this issue. Different augmentation approaches, deep network designs and generalization ability can increase the model accuracy. GAN is a novel approach for producing astonishing photorealistic graphics that emulate the content of trained datasets. GANs can effectively generate workable medical datasets that are powerful and lifelike. The datasets generated by the GAN achieved higher scores in both qualitative and quantitative assessments [188].

Several methods have been developed to reduce the extra radiation dosage related to CESM. One of the methods is to change both acquisition protocols and the imaging hardware. In 2020, Clauser et al. devised a low-dose CESM system that substitutes the traditional antiscatter grid with a software-based scatter correction approach. A significant reduction in radiation dose can be obtained by improvements in tube technology and the optimization of detector materials and structures. Using the scatter correction method, the average glandular dose for breasts of thickness 55 mm was only 1.6 mGy, which is less compared to the normal 2.2 to 2.4 mGy, suggesting reduction in average glandular dose between 21% and 48%, depending on breast thickness [189]. In conclusion, developments in hardware such as enhanced filtration, smart detection, and grid-less acquisition methods have been suggested to be beneficial for keeping consistent image quality while minimizing radiation exposure.

### 6.6. MSI

To reduce the effect of human respiration or other motion interferences in MSI, several researchers have developed and used targeted stabilizing methods. Fahad et al. used an innovative method that increased the quality of multispectral transmission images and image registration for the early assessment of BC. The study proposed a novel DL approach that combined the Vision Transformer model with Long Short-Term Memory networks to increase image registration accuracy and reduce noise in multispectral transmission images. This method enhances the SNR and grayscale resolution of the image. This method also lays the groundwork for the early identification of BC and abnormalities via improved image quality. It achieves accurate results compared to conventional methods, highlighting its innovation and robustness in handling noise and motion artifacts compared to standard optimization techniques [142]. Zhang et al. assessed image shift and rotation between frames using a high-resolution visible light camera aligned parallel to the light axis of the infrared camera. Parameter prediction was executed by adding the Kalman model. The integration of the Kalman model in the proposed method effectively reduces latency in the computed image shift and rotation parameters, enabling precise and real-time compensation [190].

To overcome spectral resolution limitations, Lanaras et al. developed a technique that achieved hyperspectral super resolution by integrating the unmixing of two input images into the pure reflectance spectra of the observed materials. Coupled matrix factorization addresses the challenges of unmixing and joint super-resolution. The formulation included adaptive spatial regularization to utilize local geometry information from the multispectral image [191]. Gewali et al. developed a complete, fully convolutional residual neural network architecture that simultaneously learns optimum multispectral bands and the transformation for hyperspectral reconstruction from multispectral signals through the analysis of extensive data sets. Yi et al. developed a unified spatial-spectral enhancement approach to simultaneously improve the resolution of MSI in both spatial and spectral domains. This establishes virtual intermediate variables that formulate the spectral observation model and a spatial observation model. It finds a solution to address spectral dictionary and abundance for the reconstruction of the necessary high-resolution images [192].

To address the deficiencies in calibration and standardization in MSI, where disparate hardware, higher costs, and large data volumes impede reproducibility, Shaikh et al. developed a system including calibration methods using an economical calibration reference made of polytetrafluoroethylene. The technique employs a hyperspectral camera along an active illumination characterized by varying spectral intensity distributions. The calibration reference was used to measure the relative reflectance of any material’s surface, independent of the spectral distribution of light and the sensitivity of the camera [193]. Ortega et al. used a calibration method that normalizes the collected hyperspectral pixels through linear scaling of the values, while also incorporating white and dark reference images [194].

## 7. Discussion and Conclusions

Advanced imaging techniques have significant potential for BC detection, as they can be coupled with various DL approaches, yielding optimal results in reduced time with high accuracy, while effectively capturing high-quality images and visualizing malignant tissue. OCT attains an average sensitivity of 93% and specificity of 98%, hence minimizing reoperations and enhancing intraoperative decision-making support. RS can identify subtypes of BC by multivariate analysis, with an accuracy of up to 99%. The integration of PAI with SVM models demonstrates a high accuracy of 95%, rendering it highly precise for diagnosis. Employing HSI with SRS attains a precision of 98% and a recall of 100%, which is outstanding and robustly endorses clinical application, while CESM demonstrates a sensitivity ranging from 95% to 98% in dense breasts, markedly surpassing that of traditional mammography. MSI demonstrates diagnostic accuracies of 99% by employing various wavelengths to identify cancerous tissue. These results indicate that this imaging methodology surpasses conventional methods regarding accuracy, sensitivity, precision, recall of lesion appearance, and early-stage identification. However, these imaging approaches include certain drawbacks that impede their clinical application. Limitations, including expensive equipment, lack of consistency in imaging methods and interpretation, prolonged acquisition and processing times, restricted penetration depth, and insufficient clinical validation, impede real-time functionality. Future developments have to concentrate on identifying alternative remedies to these issues. Linear normalization of images or the application of standardized methods might mitigate the absence of standardization. Utilizing various alternatives, such as diverse software integrated into smartphones, might diminish system costs. AI-based automated models and DL methodologies can enhance the reliability and reproducibility of these imaging techniques. Ultimately, further clinical trials and comprehensive research investigations are required to confirm the real-time accuracy across a varied patient group. With these advances, these imaging approaches possess significant potential for the precise detection of BC, and it is not contradictory to see a difference between strong performance metrics in controlled studies and the real-world problems found in clinical settings. This is just a natural result of the gap between technological innovation and clinical translation. The appropriateness of each imaging modality is contingent upon the clinical context. CESM may be a useful extra tool for screening dense breasts because it shows things more clearly than regular mammography. OCT and RS appear to be most appropriate for intraoperative applications, as both modalities can delineate tumor margins and tissue subtypes, potentially facilitating the surgeon’s decision-making process. PAI is better for characterizing lesions because it can describe both vascular and structural data. HSI and MSI could be useful for both tissue classification and early detection, but they have not been used in clinical settings yet because the equipment and requirements are too complicated and expensive. CESM is useful for dense breasts that need more than just mammography, where other methods do not work as well. The use of OCT and RS during surgery can help doctors decide what to do by helping them look at the margins. PAI, on the other hand, has some value on its own because it can show the vascular architecture of lesions. HSI and MSI could be used in the future to find and classify tissues early and cheaply, since both current methods are limited by workflow and cost. These methods show how new ideas can be slowly added to the current ways of diagnosing and treating breast cancer to make them better. In this way, there is no one best method; instead, each method can be used for a certain purpose. A wider range of methods will mean finding a balance between what is possible, what is available, what is comfortable for the patient, and what is available in healthcare. It is important to note that the imaging methods on this list are important diagnostic tools that could help with finding, staging, and guiding surgery. Nonetheless, the definitive diagnosis of breast cancer and the identification of its molecular characteristics remain contingent upon morphological and immunohistochemical analyses of tumor tissue. Moreover, the high sensitivity, specificity, and accuracy values reported in numerous studies reflect performance under controlled research conditions, typically involving well-defined cohorts and optimized imaging protocols. These results represent the technical potential of the modalities rather than standard clinical outcomes. These values are not introduced in vain; they provide standards of feasibility and indicate which imaging methods are most likely to be successfully applied in larger trials. Future multicenter studies will be required to ascertain the comparisons between these research-level outcomes and actual clinical performance. Along with these new imaging technologies, there has been a lot of growth in AI-based diagnostic models and radiomics applications in the last few years. These use quantitative features of medical images to improve classification, prediction, and risk stratification. These methods have demonstrated superior efficacy in lesion detection, subtype classification, and outcome prediction, and are presently under investigation alongside novel imaging modalities such as HSI, PAI, and CESM. This article does not provide a comprehensive review of AI and radiomics; however, their integration with rapidly advancing imaging technologies holds significant potential to enhance diagnostic accuracy, reduce interpretative variability, and facilitate personalized treatment approaches. Integrating these novel imaging modalities into standard clinical practices is likely to be a task-specific endeavor rather than a universal one. There are still problems with clinical adoption, such as the high cost of equipment, the lack of standardization, and the depth of penetration. However, a number of possible solutions are starting to become clear. Multicenter research and established imaging protocols can facilitate further standardization, while costs may be reduced through the miniaturization and integration of the device with existing technologies, such as portable or smartphone-based systems. AI-powered automated models can also help with reproducibility, make it easier to analyze images, and reduce the amount of work that the operator has to do. All of these advances together could help close the gap between what is possible in research and what is done in real life.

## Figures and Tables

**Table 1:** Case Studies on BC Detection Using OCT.

Study By	Imaging Modality/Method Used	Datasets Used	Results
Dhiman et al. [92]	FFOCT + Ensemble Classifier based on Technique for Order of Preference by Similarity to Ideal Solution	OCT images of 48 patients of 35–60 years old	Precision = 92.1%, Recall = 92.1%, Accuracy = 92.3%, F1-score = 0.921
Zhang et al. [93]	Dynamic FFOCT + Swin Transformer	13,497 patches of 129 patients	Accuracy = 97.62%, Sensitivity = 96.88%, Specificity = 100%
Sanderson et al. [94]	In vivo OCT	16 breast-conserving surgery patients + 139 in vivo OCT scans	Co-registration rate 78%
Yang et al. [95]	High-Res FFOCT + Dynamic Imaging	314 tissue specimens, 173 breast biopsies, and 141 lymph nodes	DCI Sensitivity = 88.6%, Specificity = 95.1% FFOCT Sensitivity = 85.6%, Specificity = 85.4%
Simon et al. [96]	FFOCT + Dynamic Cell Imaging	217 biopsies of 152 patients	Sensitivity = 77%, Specificity = 64% PPV = 74% NPV = 75%
Gubarkova et al. [97]	3D CPOCT + depth-resolved approach + cross-polarization channels	68 excised human breast specimens	Accuracy = 91% to 99%, Sensitivity = 96% to 98%, Specificity = 87% to 99%
Faragalla et al. [98]	OCT	175 samples of tissue were obtained from 40 specimens of breast tissue	Detected that 30% of the samples
Levy et al. [99]	WFOCT + CNN	585 Wide Field OCT margin scans	Sensitivity = 93% Specificity = 98% AUROC = 0.976
Sun et al. [100]	PSOCT + GANs	22,072 PS-OCT images	AUC, DOPU = 0.979 Phase retardation = 0.952
Basu et al. [101]	PS-FFOCT + TOPSIS	220 sample images	Precision = 94.8%, Recall = 92.5% F-score = 93.7% MCC = 82.3%

**Table 2:** Case Studies on BC Detection Using PAI.

Study By	Imaging Modality	Datasets Used	Results
Tong et al. [112]	Panoramic PACT	78 breasts of 39 patients	AUROC = 0.89
G. Li et al. [113]	Dual modal photoacoustic-ultrasound examination	324 patients	Nomogram, AUROC = 0.868 on test set AUC = 0.906 on training set
Huang Z et al. [114]	PAI + univariate and multivariate logistic regression	group of 317 individuals	AUC = 0.815 to 0.950 CI = 95%
Li et al. [115]	PAI + Murine model	50 xenografts	Accuracy = 72% Sensitivity = 66% Specificity = 78%
Rodrigues et al. [116]	PAI + SVM Algorithms	Xenografts from 5 mice	Accuracy, SVM-RBF = 95.2% SVM Polynomial = 99.5% SVM-Linear = 80.3%
Huang et al. [117]	PAI Radiomics + Multivariate logistic regression	359 patients with BC	AUC = 0.899
Guoqiu et al. [118]	PAI + ResNet50	334 patients	Sensitivity = 78.6% Specificity = 87.2% Accuracy = 83.6%
Zhang et al. [119]	Multispectral PAI	Formalin-fixed paraffin-embedded blocks of both healthy and cancerous human breast tissue	Mean Correlation, Cancer tissue = 0.762–0.954 Healthy tissue = 0.801 to 0.967
Wang et al. [120]	Photoacoustic/ultrasound imaging	45 patients (17 had breast intraductal neoplasm, 26 had non-intraductal malignant neoplasm)	Sensitivity = 90% Specificity = 87.5%
Guoqiu et al., [121]	BI-RADS and PAI Radiomics	119 women patients	AUC = 0.926 on test set AUC 0.925 on the training set

**Table 3:** Studies on BC Detection Using CESM.

Study By	Imaging Modality	Datasets Used	Results
Jailin et al. [129]	CESM + YOLO architecture	1673 patients and 7443 CESM pictures	AUROC = 0.964 Able to detect 90% of cancer
Bouzarjomehri et al. [130]	CESM + Digital mammography	CDD-CESM dataset of 326 patients	Accuracy = 98.85% CM = 97.47%
Mao et al. [131]	CESM + CBAM-based Xception,	CESM images of 1239 patients	Sensitivity = 84.8% Specificity = 100% Accuracy = 89.1%
Chen et al. [132]	MDCS + CESM	large multicentre cohort of CESM scans of 1903 females	Classification AUC = 0.912
Miller et al. [133]	Quantitative CESM + Logistic regression	159 suspicious breast findings	Accuracy = 71.5% AUC-ROC = 0.81
Moffa et al. [134]	CESM + breast ultrasound	group of 51 patients with 65 breast lesions	Accuracy = 87.7% Sensitivity = 93.5% Specificity = 79.4%
Lin et al. [135]	CESM Radiomics + ANOVA and Multivariate logistic regression	139 patients	AUC = 0.940 Confidence Interval = 95%
Zheng et al. [136]	RefineNet, Xception + Pyramid pooling module	1912 patients	AUC = 0.940
Gouda et al. [137]	CESM vs. MRI	60 women with BC	CESM Accuracy = 95% Sensitivity = 97% Specificity = 67% MRI Accuracy = 94% Sensitivity = 99% Specificity = 33%
Song et al. [138]	CESM + GAN-based image fusion module and a Res2Net-based classification module	760 images of the CESM of 95 patients	Accuracy = 94.784% Precision = 95.016% Recall = 95.912%
Pediconi et al. [139]	CESM + HCCM	205 patients exposed to CESM	Sensitivity = 96–97% Specificity = 84–87% Accuracy = 93–95%
Sun et al. [140]	CESM + LASSO, Random Forest	157 women and 161 breast lesions	LASSO, Accuracy = 89.5% Sensitivity = 89.1% Specificity =90.8% RF, Accuracy = 88% Sensitivity = 87.8% Specificity = 88.6%
Song et al. [141]	CESM + Res2Net50	760 CESM images of 95 patients	Sensitivity = 96.350% Specificity = 96.396% Accuracy = 96.591%

## Data Availability

The data presented in this study are available in this article upon considerable request to the corresponding author (H.-C.W.).

## Abbreviations

The following abbreviations are used in this manuscript:

BC	Breast cancer
DL	Deep Learning
ML	Machine Learning
AI	Artificial Intelligence
OCT	Optical coherence tomography
RS	Raman Spectroscopy
PAI	Photoacoustic Imaging
HSI	Hyperspectral Imaging
CESM	Contrast-Enhanced Spectral Mammography
MSI	Multispectral Imaging
CNN	Convolutional Neural Network
AUC	Area under the Curve
AUC-ROC	Area under the Receiver Operating Characteristic Curve
RMSE	Root Mean Square Error
MCC	Matthews Correlation Coefficient
DOPU	Degree of Polarization Uniformity
SERS	Surface-Enhanced Raman Spectroscopy
PCA	Principle Component Analysis
SVM	Support Vector machine
MDCS	Multiprocess Detection and Classification System
GANs	Generative Adversarial Networks
HCCM	High-Concentration Iodinated Contrast Medium
LASSO	Least Absolute Shrinkage and Selection Operator
H&E	Hematoxylin & Eosin
PAT	Photoacoustic Tomography
SNR	Signal-to-Noise Ratio
ROI	Region of Interest
DNN	Deep Neural Network
ISPIM	Inverted Selective Plane Illumination Microscopy
FAPS	Fully Automated Pipeline system
